# An integrated path for spatial capture–recapture and animal movement modeling

**DOI:** 10.1002/ecy.3473

**Published:** 2021-09-30

**Authors:** Brett T. McClintock, Briana Abrahms, Richard B. Chandler, Paul B. Conn, Sarah J. Converse, Robert L. Emmet, Beth Gardner, Nathan J. Hostetter, Devin S. Johnson

**Affiliations:** ^1^ Marine Mammal Laboratory NOAA‐NMFS Alaska Fisheries Science Center Seattle Washington USA; ^2^ Department of Biology University of Washington Life Sciences Building, Box 351800 Seattle Washington USA; ^3^ Warnell School of Forestry and Natural Resources University of Georgia 180 E. Green St. Athens Georgia USA; ^4^ U.S. Geological Survey Washington Cooperative Fish and Wildlife Research Unit School of Environmental and Forest Sciences & School of Aquatic and Fishery Sciences University of Washington Box 355020 Seattle Washington USA; ^5^ Quantitative Ecology and Resource Management University of Washington Seattle Washington USA; ^6^ School of Environmental and Forest Sciences University of Washington Seattle Washington USA; ^7^ Washington Cooperative Fish and Wildlife Research Unit School of Aquatic and Fishery Sciences University of Washington Seattle Washington USA

**Keywords:** animal movement, density and distribution, integrated population model, mark–recapture, movement ecology, population dynamics, population ecology, spatial capture–recapture

## Abstract

Ecologists and conservation biologists increasingly rely on spatial capture–recapture (SCR) and movement modeling to study animal populations. Historically, SCR has focused on population‐level processes (e.g., vital rates, abundance, density, and distribution), whereas animal movement modeling has focused on the behavior of individuals (e.g., activity budgets, resource selection, migration). Even though animal movement is clearly a driver of population‐level patterns and dynamics, technical and conceptual developments to date have not forged a firm link between the two fields. Instead, movement modeling has typically focused on the individual level without providing a coherent scaling from individual‐ to population‐level processes, whereas SCR has typically focused on the population level while greatly simplifying the movement processes that give rise to the observations underlying these models. In our view, the integration of SCR and animal movement modeling has tremendous potential for allowing ecologists to scale up from individuals to populations and advancing the types of inferences that can be made at the intersection of population, movement, and landscape ecology. Properly accounting for complex animal movement processes can also potentially reduce bias in estimators of population‐level parameters, thereby improving inferences that are critical for species conservation and management. This introductory article to the Special Feature reviews recent advances in SCR and animal movement modeling, establishes a common notation, highlights potential advantages of linking individual‐level (Lagrangian) movements to population‐level (Eulerian) processes, and outlines a general conceptual framework for the integration of movement and SCR models. We then identify important avenues for future research, including key challenges and potential pitfalls in the developments and applications that lie ahead.

## Introduction

Understanding the processes that influence species abundance, density, demographic rates, spatial distribution, and habitat selection are central goals in ecology and fundamental to biodiversity conservation (Williams et al. 2002, Manly et al. 2007, MacKenzie et al. 2018). Driven by a need to quantify these processes, both spatial capture–recapture (SCR; Royle et al. 2013*b*) and animal movement modeling (Hooten et al. 2017) have independently seen widespread adoption and advancement in recent decades. Unifying movement modeling—which has traditionally focused at the level of the individual to understand animal movement and space use—and SCR modeling—which has primarily been concerned with population‐level processes such as abundance and demographic rates—offers incredible potential to provide new insights at the intersection of population, movement, and landscape ecology (Morales et al. 2010, Matthiopoulos et al. 2015, Ovaskainen et al. 2016, Royle et al. 2018).

SCR and the burgeoning field of movement ecology (Nathan et al. 2008) offer complementary toolkits. Royle et al. (2013*b*:426–429,534–535) recognized this unrealized potential and identified the integration of animal movement and SCR models as “one of the most exciting areas” of future methodological advancement in these fields. By integrating movement models that can more realistically portray how animals use space, inference from SCR models can be improved (Ovaskainen 2004, Tufto et al. 2012, Royle et al. 2013*a*, *2013a*, *2013c*, 2016, Borchers et al. 2014). For example, properly accounting for complex animal movement processes can potentially reduce bias of population parameters estimated from SCR (Borchers et al. 2014, Royle et al. 2016). Given the reliance on accurate population parameters for species conservation and management, developing methods to improve these estimators is critical.

**Fig. 1:**
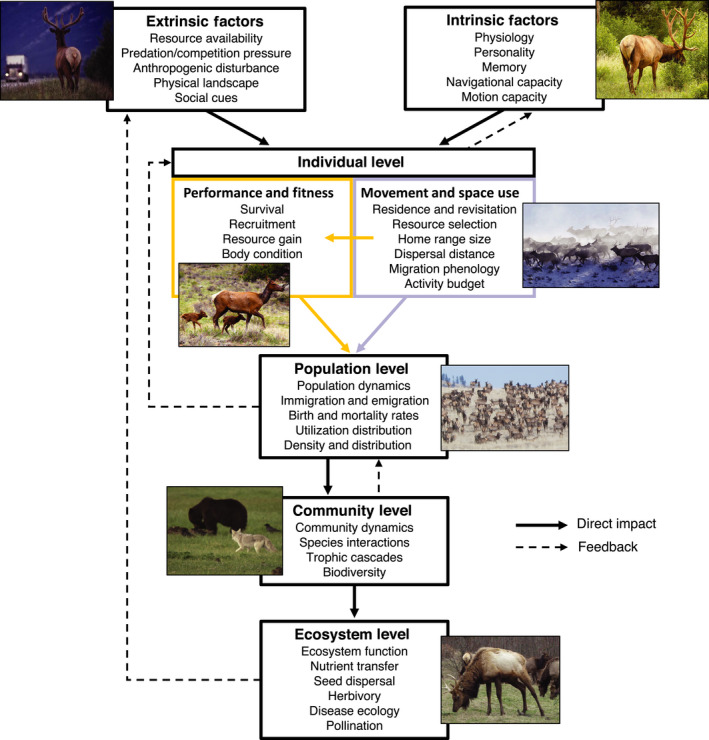
A framework for developing hypotheses that could be empirically tested with integrated animal movement and spatial capture–recapture models. Intrinsic and extrinsic factors have direct effects on individual movements and performance, which in turn impact population‐level and higher‐order processes. Orange and purple coloring indicates processes that are typically investigated using capture–recapture or movement modeling, respectively.

More interesting still, the integration of movement modeling and SCR methods offers new opportunities to link individual animal movement behavior (e.g., dispersal, migration, habitat selection) to population dynamics, density, and distribution (Dunning et al. 1995, Nathan et al. 2008, Morales et al. 2010, Royle et al. 2013*b*: Chapter 16, Matthiopoulos et al. 2015, Spiegel et al. 2017). For example, the way an individual animal chooses to move and behave in response to extrinsic factors (e.g., predation risk) affects its survival and recruitment; when this process is aggregated over many individuals, population‐level parameters such as density and distribution emerge, which in turn affect higher‐order processes such as community assemblage and ecosystem function (Fig. 1). Yet despite the clear and important connections between individual‐level processes and the dynamics of populations, communities, and ecosystems, establishing these linkages quantitatively remains a key challenge for ecologists. Integrating animal movement modeling and SCR could provide a much‐needed analytical framework for empirically testing such hypotheses.

This introductory article to the Special Feature briefly reviews SCR and animal movement modeling (see *Looking Back: How Did We Get Here?*) and establishes a common notation to facilitate integration of these approaches (Table 1). We then provide motivation for their integration and outline a general conceptual framework for incorporating explicit animal movement processes into SCR models (see *Integrating Movement and Spatial Capture–Recapture*). Central to this framework is conditioning the detection process in SCR models on the dynamic location of animals instead of a static “home range” (or “activity”) center. We conclude by identifying key challenges, highlighting future directions, and positioning the contributions of the Special Feature relative to the exciting research and development that lies ahead (see *Looking Forward: Where Are We Going?*).

**Table 1:** Statistical notation for integrated movement and spatial capture–recapture (SCR) modeling.

Notation	Definition
General	
*N*	Number of individuals in the population (i.e., abundance), with index i=1,…,N
*T*	Number of time points in the detection or movement process, with index t=1,…,T (in units of interest). The time interval between points is t‐1 and t is denoted as Δt
θ	Vector of all unknown parameters to be estimated. This includes observation and movement process parameters, as well as any other demographic parameters commonly included in capture–recapture models (e.g., survival)
$\left[\cdot \right]$	Bracket notation for probability density or mass function. For example, $\left[y|\theta \right]$ denotes the conditional probability of the data $\left(y\right)$, given the parameters $\left(\theta \right)$
Movement	
μ	True individual location (i.e., position), typically a 2×1 vector for true position in two‐dimensional continuous space
M	Spatial support for the true position process (i.e., μ∈M), typically $M\subset R^{2}$ in two‐dimensional continuous space
ε	Error term describing the stochastic component of the true position process $\left(\mu \right)$, for example, $\varepsilon ∼N\left(0,\sum \right)$ or $\varepsilon ∼N\left(0,\sigma^{2}I\right)$, where I is the identity matrix
$\sigma^{2}$	Variance component associated with the true position process $\left(\mu \right)$
∑	Covariance matrix associated with the true position process $\left(\mu \right)$
s	Expected value for the true position process $\left(\mu \right)$ over a fixed interval of time $\left(s\in M\right)$. This corresponds to the activity center in standard SCR models
u	Observed individual location (e.g., from telemetry or opportunistic data), typically a 2×1 vector for observations in two‐dimensional continuous space. If a location is observed without measurement error, then u=μ
$\sigma_{u}^{2}$	Measurement error variance component associated with observed locations $\left(u\right)$
$\sum_{u}$	Measurement error covariance matrix associated with observed locations $\left(u\right)$
SCR	
J	Number of detectors (e.g., camera traps, acoustic receivers), with index j=1,…,J
x	Location of detector, typically a 2×1 vector in two‐dimensional space
y	Spatial detection (or encounter) history
$\sigma_{\text{det}}^{2}$	Variance component associated with the detection process. The detection process is assumed to be a function of x and μ (or s)

Notes

Data and latent variables are indicated by Roman letters, and parameters are indicated by Greek letters (with the exception of N and μ for historical reasons). Bold lowercase letters indicate vectors, and bold uppercase letters indicate matrices. For simplicity, possible subscripts for individual $\left(i\right)$, time $\left(t\right)$, or detector $\left(j\right)$ are omitted.

## Looking Back: How Did We Get Here?

### Spatial capture–recapture

Capture–recapture is one of the oldest and most popular approaches to inference in animal population ecology (Williams et al. 2002). However, despite the fact that capture–recapture data include valuable information about animal space use, conventional capture–recapture models have historically ignored the location of captured individuals for the estimation of abundance (or density) and related demographic parameters (but see, e.g., Brownie et al. [1993], Schwarz et al. [1993], Ovaskainen [2004], Lagrange et al. [2014], Bishop and Bernard [2021]). This has many disadvantages, including a poorly defined effective sampling area for inferences about population density, potential bias induced by unmodeled heterogeneity in detection probability attributable to individual space use, and an inability to link movement, space use, or resource selection to population dynamics (Royle et al. 2013*b*). Efford (2004) recognized this as a missed opportunity and proposed spatially explicit models to capitalize on the spatial information contained in capture–recapture data. Spatial capture–recapture was soon expanded upon by Borchers and Efford (2008), Royle and Young (2008), Gardner et al. (2009), and many others towards the development of an inferential framework that has revolutionized the field of capture–recapture over the past decade. We assume the reader already has some basic understanding of (spatial) capture–recapture and only highlight elements of particular relevance to the integration of SCR and movement modeling. Royle et al. (2013*b*) provide a thorough introduction to the basic SCR model and its early extensions, and Borchers and Fewster (2016) and Royle et al. (2018) review more recent SCR developments.

#### SCR models

The basic SCR model for population abundance (or density) assumes a closed population where the “activity” (or “home range”) center of each individual resides within a prescribed area called the state space $\left(M\right)$ during the period of sampling. The activity center can be interpreted as the expected location about which an individual uses space during the study period. Individuals can either be naturally (e.g., distinct pelage patterns) or artificially marked at first capture. For ease of exposition, we will assume detections of marked individuals occur in discrete time over T sampling intervals (or occasions) across a fixed array of J traps, but SCR models can accommodate other types of study designs (e.g., search–encounter data; Efford [2011], Royle et al. [2011]) or be formulated in continuous time (Ovaskainen 2004, Borchers et al. 2014, Dorazio and Karanth 2017).

Central to SCR is relating the spatial encounter history for each individual $\left(y_{i}\right)$ to its activity center $\left(s_{i}\right)$, where the density and distribution of activity centers are assumed to arise from a spatial point process (Borchers and Efford 2008, Royle et al. 2013*b*: Chapter 11). Heuristically, we have

(1) $$\left[y|s,N,\theta \right]=\underset{\text{SCR model}}{\underset{︸}{\left[y|s,\theta \right]}}\overset{\text{point process model}}{\overset{︷}{\left[s,N|\theta \right]}},$$

where $\left[y|s,\theta \right]$ is the observation model for the spatial encounter history data, $\left[s,N|\theta \right]$ is the spatial point process model for the activity centers, N is the size of the population within M, and θ is a vector of the observation and point process parameters embedded in these two model components. Note that the activity centers are not observed, and these latent variables must therefore be marginalized out of the conditional likelihood by integrating over the support of the state space $\left(s\in M\right)$ when fitting models with likelihood‐based methods (see *Model fitting*).

Standard SCR models often assume encounter history data are the outcomes of independent Bernoulli trials $\left(y_{\mathrm{ijt}}\in \left\{0,1\right\}\right)$, where detection probability $p_{\mathrm{ij}}=\text{Pr}\left(y_{\mathrm{ijt}}=1\right)$ for individual i at trap j is a function of the location of the activity center $s_{i}$ and the location of the trap $x_{j}$ (Borchers and Efford 2008, Royle et al. 2009). For example,

(2) $$p_{\mathrm{ij}}=p_{0}\exp\left(‐\frac{{∥x_{j}‐s_{i}∥}^{2}}{2\sigma_{\text{det}}^{2}}\right)$$

is based on the kernel of a Gaussian probability density, where $∥x_{j}‐s_{i}∥$ is Euclidean distance and $p_{0}$ is the baseline detection probability at distance zero. Typically denoted as σ in the SCR literature, the scale parameter (σ_det_) can be proportional to home range size when activity centers are static, home ranges are symmetric, and space within home ranges is used independently. The implicit assumption is that animals use space according to a bivariate normal distribution centered at $s_{i}$, and, regardless of their location in space or local habitat conditions, space use for all individuals is therefore symmetric with circular contours of usage intensity. Binomial, Poisson, or other observation distributions can also be used for modeling the detection process (Royle et al. 2013*b*: Chapters 2 and 9), but these implicit assumptions about animal movement and space use remain unchanged.

**Fig. 2:**
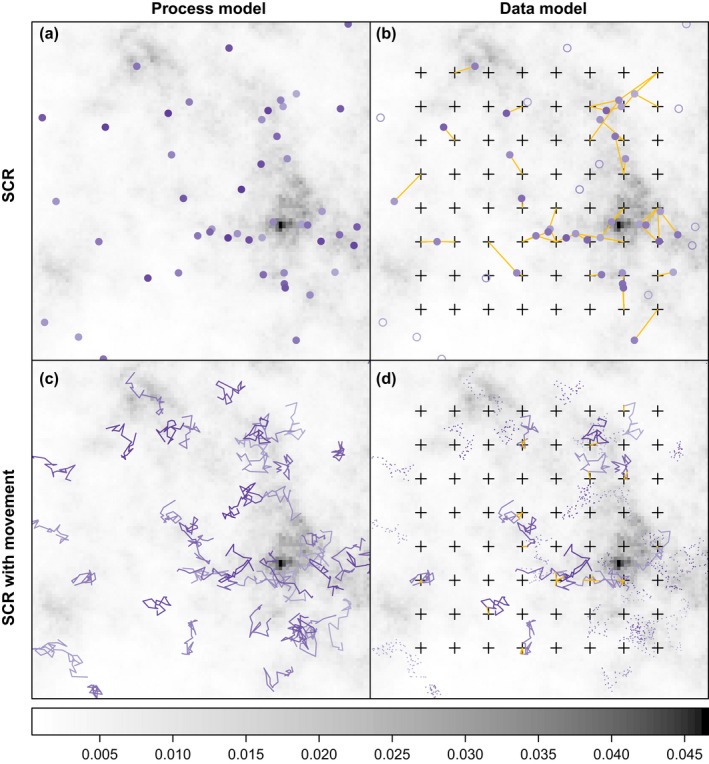
The standard spatial capture–recapture (SCR) point process model (a) describes the abundance and distribution of animal activity centers (purple circles). The standard SCR observation model (b) describes how detection probability decreases with distance between activity centers and traps (black crosses). When integrating movement and SCR models, the process model (c) describes the abundance and location of individuals through time (purple lines), and the observation model (d) describes how detection probability decreases with distance between individual locations and traps. In this example, the movement trajectories follow a biased random walk with a static center of attraction for each individual. Abundance and distribution of activity centers in (a) and initial locations (or centers of attraction) in (c) are governed by an intensity function (represented here by the density surface, where darker shades indicate higher density), which can depend on covariates and spatial random effects. In this simulation, individuals with filled circles (b) or solid lines (d) were detected at the traps indicated by the orange line segments.

For the spatial point process model describing the density and distribution of activity centers, the most common formulations are homogeneous Poisson or binomial point processes (Borchers and Efford 2008, Royle et al. 2013*b*: Chapter 5), typically partitioned as $\left[s,N|\theta \right]=\left[s|N,\theta \right]\left[N|\theta \right]$, where $\left[s|N,\theta \right]=\prod_{i=1}^{N}\left[s_{i}|\theta \right]$. Under the homogeneous Poisson model, the number of activity centers in the state space (i.e., N) is modeled as $N|\lambda ∼\text{Poisson}\left(\lambda ∥M∥\right)$, where the intensity parameter λ is the density of activity centers, $E\left(N\right)=\lambda ∥M∥$ is the expected abundance, and ∥M∥ is the area of the state space. Another parameterization is $N|\psi ∼\text{Binomial}\left(M,\psi \right)$, where $E\left(N\right)=\psi M$, *M* ≫ *N* is a known index—typically established using an approach known as parameter‐expanded data augmentation (Royle et al. 2007, Royle and Dorazio 2012)—for the maximum number of possible activity centers in the state space, and ψ is the probability that any given one of these M possible activity centers belongs to one of the N individuals that are members of the population. In either case, the N activity centers are assumed to be distributed uniformly within the state space, $s_{i}∼\text{Uniform}\left(M\right)$ for i=1,…,N, regardless of habitat conditions or behaviors such as territoriality. These uniformity assumptions can be relaxed by using inhomogeneous point process models (see Fig. 2) based on suitable habitat masks or other habitat covariates (e.g., habitat type, elevation) to facilitate inferences about factors driving population density and distribution (Borchers and Efford 2008, Royle et al. 2013*b*: Chapter 11). For example, under the Poisson model, we could instead have

$$N|\beta ∼\text{Poisson}\left(\lambda_{0}\int_{M}\lambda \left(s\right)ds\right)$$

and

$$\left[s_{i}|\beta \right]=\frac{\lambda \left(s_{i}\right)}{\int_{M}\lambda \left(z\right)dz},$$

where

$$\lambda \left(s\right)=\exp\left(\sum_{k=1}^{K}\beta_{k}c_{k}\left(s\right)\right)$$

is the intensity function at location s, $\lambda_{0}=\exp\left(\beta_{0}\right)$ is the baseline intensity rate, $c_{k}\left(s\right)$ is the value of the kth habitat covariate at location s, and $\beta_{k}$ the corresponding coefficient.

#### Extensions

There has been a methodological explosion of SCR models over the past decade (Royle et al. 2013*b*, Borchers and Fewster 2016, Royle et al. 2018). Extensions have been developed for open populations (Gardner et al. 2010, Schaub and Royle 2014, Glennie et al. 2019, Efford and Schofield 2020), resource selection (Royle et al. 2013*c*), landscape connectivity (Royle et al. 2013*a*), territorial species (Reich and Gardner 2014), attractions between individuals (McLaughlin and Bar 2020), integrated population models (Chandler and Clark 2014), passive acoustics (Efford et al. 2009, Kidney et al. 2016, Measey et al. 2017), and detections of unmarked or telemetered individuals (Chandler and Royle 2013, Sollmann et al. 2013, Efford and Hunter 2018). Instead of directly modeling the animal movement process, existing SCR models integrating resource selection, landscape connectivity, or spatial interactions rely on Eulerian formulations of space usage based on inclusion of habitat covariates or cost functions in the detection function (see Eq. 2) or an inhomogeneous point process model for static activity centers (Royle et al. 2018). Although there has already been some pioneering work towards integrating movement processes into SCR, these have largely focused on dynamic activity centers that change locations as simple random walks between primary sampling occasions in open population models (Ergon and Gardner 2014, Schaub and Royle 2014, Glennie et al. 2019, Efford and Schofield 2020) or to address transience in closed population models (Royle et al. 2016). The potential advantages of SCR models incorporating explicit and more realistic movement processes have been repeatedly noted (Royle et al. 2013*b*, Borchers et al. 2014, Borchers and Fewster 2016, Royle et al. 2018, Distiller et al. 2020), but progress has nevertheless been limited. In the sections that follow, we highlight how recent developments in animal movement modeling can provide the basic building blocks from which more realistic movement processes can be incorporated into SCR models.

### Movement modeling

The field of movement ecology holds great potential for exciting new inferences about space use, resource selection, landscape connectivity, dispersal, population dynamics, behavior, fitness, gene flow, and physiology (Nathan et al. 2008, Morales et al. 2010, Mueller et al. 2011, Matthiopoulos et al. 2015, Dickson et al. 2019). Largely owing to technological advances in animal‐borne telemetry (Cooke et al. 2004, Cagnacci et al. 2010, Hussey et al. 2015, Kays et al. 2015), remote sensing (Gao 2002), and computing power, animal movement modeling has experienced an explosion of development and application in recent years (e.g., see reviews by Thurfjell et al. [2014], Hooten et al. [2017], Patterson et al. [2017]). Like SCR, most animal movement models rely heavily on concepts from spatial statistics (e.g., point process models; Hooten et al. 2017: Chapter 4) and time series analysis (e.g., random walk models; Hooten et al. 2017: Chapter 3). These models are typically informed by animal‐borne telemetry data, but they have been developed for other types of data including capture–recapture (Ovaskainen 2004, Ovaskainen et al. 2008) and passive acoustics (Pedersen and Weng 2013, Winton et al. 2018).

In *Spatial capture–recapture*, we noted that SCR models typically use Poisson or binomial point processes for describing the number and spatial distribution of activity centers (Borchers and Efford 2008, Royle et al. 2013*b*: Chapter 11). In the animal movement literature, resource selection functions (RSFs) are popular spatial point process models for investigating space use based on habitat composition and inferring (steady‐state) utilization distributions from telemetry data (Manly et al. 2007). However, as telemetry data have continued to increase in temporal resolution, there has been a need to extend RSFs to account for autocorrelation in the time series, including step selection functions (Fortin et al. 2005, Forester et al. 2009, Thurfjell et al. 2014, Avgar et al. 2016, Wang et al. 2019), Brownian bridges (Horne et al. 2007, Kranstauber et al. 2012, Byrne et al. 2014), and, more generally, spatio‐temporal point process models (Johnson et al. 2013, Brost et al. 2015, Hooten et al. 2017: Chapter 4) that distinguish resource availability from selection using an explicit model for animal movement. There has also been some interesting recent work attempting to formulate (Lagrangian) movement models—describing the microscopic rules of individual animal movement—that, when scaled up in time and space, give rise to the expected (Eulerian) utilization distribution—describing the macroscopic distribution of a population—in a coherent framework (Potts et al. 2014, Ovaskainen et al. 2016: Chapter 2, Michelot et al. 2019a, 2019b, *2019a*, *2019b*, Potts and Schlägel 2020).

Lagrangian movement models are typically based on variations of random walk time series models formulated in either discrete or continuous time. As with SCR, applications of animal movement models have historically been dominated by discrete‐time formulations (Hooten et al. 2017: Chapter 5). While continuous‐time models can bring key advantages (see *Looking Forward: Where Are We Going?*) and are arguably a more natural way to think about animal movement, discrete‐time models have generally been considered more accessible to practitioners (McClintock et al. 2014). The prevalence of discrete‐time models in both SCR and animal movement applications may be due in part to them being more intuitive to ecologists less familiar with stochastic differential equations and instantaneous rate parameters. For ease of exposition, we therefore primarily focus on discrete‐time formulations to demonstrate some fundamental building blocks of animal movement models, but there are continuous‐time analogues for each component described in *Random walks* (Dunn and Gipson 1977, Blackwell 2003, Johnson et al. 2008, Ovaskainen and Crone 2009, Calabrese et al. 2016, Gurarie et al. 2017, Hooten et al. 2017: Chapter 6). An intuitive way to think about continuous time movement is to imagine a discrete‐time model with infinitesimally small increments $\left(\Delta t\right)$ between times t‐1 and t (i.e., Δt→0).

#### Random walks

**Fig. 3:**
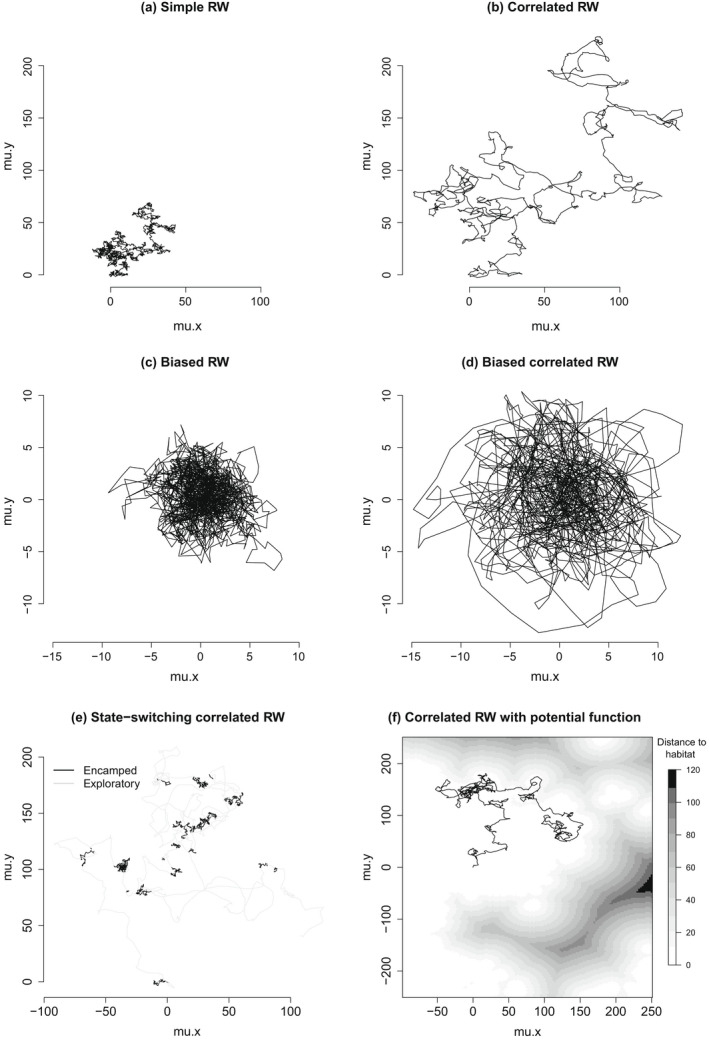
Discrete‐time random walks (RW) initiated at the origin with *T* = 1,500 and $\sigma^{2}=1$. Examples include (a) simple RW, (b) correlated RW with γ=0.7 and β=0, (c) biased RW with central tendency where $a=\left(0,0\right)$ and ρ=0.1, (d) biased correlated RW, (e) state‐switching correlated RW with $\sigma_{1}^{2}=1$ and $\sigma_{2}^{2}=5$, and (f) correlated RW with potential function constraining movement to preferred habitat (in white) with δ=‐50,000.

Most discrete‐time formulations are extensions of the simple random walk model (Fig. 3a):

(3) $$\mu_{t}=\mu_{t‐1}+\varepsilon_{t},$$

for t=1,…,T, where $\mu_{t}$ is the animal location at time t (typically measured in continuous two‐dimensional space) and the errors $\varepsilon_{t}∼N\left(0,\sum \right)$ describe the dispersion process (see Table 1). Equivalently, we have $\mu_{t}∼N\left(\mu_{t‐1},\sum \right)$. It is common to assume the errors are symmetric, that is, $\sum =\sigma^{2}I$, where I is the identity matrix. It is straightforward to extend the simple random walk to a correlated random walk with short‐term directional persistence (Fig. 3b):

(4) $$\mu_{t}=\mu_{t‐1}+R\left(\mu_{t‐1}‐\mu_{t‐2}\right)+\varepsilon_{t},$$

where the rotational component

(5) $$R=\gamma \left[\begin{array}{cc} \text{cos}\left(\beta \right) & ‐\text{sin}\left(\beta \right)\\ \text{sin}\left(\beta \right) & \text{cos}\left(\beta \right) \end{array}\right]$$

controls the degree of correlation in the movement path with mean turn angle $\beta \in \left[‐\pi ,\pi \right)$ and damping parameter $\gamma \in \left[0,1\right]$ (Jonsen et al. 2005). A turn angle of zero indicates no change in direction between successive time steps, and γ=0 reduces Eq. 4 to a simple random walk (Eq. 3). A central tendency can be incorporated using a biased random walk with attraction towards a particular location (Fig. 3c):

(6) $$\mu_{t}=\mu_{t‐1}+B\left(a‐\mu_{t‐1}\right)+\varepsilon_{t},$$

where B=ρI, $\rho \in \left[0,1\right]$ controls the strength of attraction towards location a, and ρ=0 reverts Eq. 6 back to a simple random walk (Eq. 3). With ρ>0, the individual’s path is ensured to be stationary over time (i.e., the animal must move toward a eventually). Combining Eqs. 4 and 6 yields a biased correlated random walk (Fig. 3d):

(7) $$\mu_{t}=\mu_{t‐1}+B\left(a‐\mu_{t‐1}\right)+R\left(\mu_{t‐1}‐\mu_{t‐2}\right)+\varepsilon_{t}.$$

Animal movement behavior is of course more complicated than these relatively basic random walks, and there have been many recent extensions to incorporate more realism. A popular approach is to incorporate multiple modes (or “states”) of movement that reflect different behaviors, such as “encamped” or “exploratory” movement (Franke et al. 2004, Morales et al. 2004), multiple centers of attraction (McClintock et al. 2012, Pirotta et al. 2018), or group‐dynamic movement (Langrock et al. 2014). For example, behavioral state‐switching can be incorporated by conditionally specifying the mode of movement at each time step based on the underlying state $\left(m_{t}\right)$ of the animal at time t (Fig. 3e):

(8) $$\mu_{t}=\left\{\begin{array}{cc} \mu_{t‐1}+\varepsilon_{t} & \text{if}\;m_{t}=1\\ \mu_{t‐1}+R\left(\mu_{t‐1}‐\mu_{t‐2}\right)+\varepsilon_{t} & \text{if}\;m_{t}=2 \end{array}\right.,$$

where $m_{t}=1$ indicates the animal is in an “encamped” state (i.e., smaller displacement with no directional persistence), $m_{t}=2$ indicates an “exploratory” state (i.e., larger displacement with directional persistence), $\varepsilon_{t}∼N\left(0,\sigma_{m_{t}}^{2}I\right)$, $\sigma_{m_{t}}$ is the (state‐dependent) dispersion parameter, and $\sigma_{1}\;<\;\sigma_{2}$. Individual or temporal heterogeneity can also be incorporated into movement parameters using explanatory covariates (e.g., age, sex, reproductive status; Grecian et al. 2018, Carter et al. 2020) as is commonly done for survival and other demographic parameters in capture–recapture analyses (White and Burnham 1999).

Habitat also plays an important role in animal movement. Potential functions provide a straightforward mathematical framework for movement along environmental gradients (Preisler et al. 2004, Brillinger et al. 2012, Hooten et al. 2017: Chapter 6). As an illustration, suppose the movement of an animal is constrained to be within a particular habitat type (e.g., island, body of water, valley within mountainous terrain). Much like a marble rolling along a hilly surface, potential functions can steer movements towards (or away from) particular habitat features based on their gradients:

(9) $$\mu_{t}=\mu_{t‐1}+D\nabla c\left(\mu_{t‐1}\right)+\varepsilon_{t},$$

where $c\left(\mu_{t‐1}\right)$ is the habitat covariate evaluated at location $\mu_{t‐1}$, ∇ is the gradient operator, D=δI, and δ controls the movement response to this habitat gradient. The gradient is a vector field of partial derivatives pointing in the direction of the greatest rate of increase in the habitat covariate, with length corresponding to the rate of increase in that direction (Dawber 1987: Chapter 4). Thus δ<0 indicates the animal generally moves to areas with lower values of the covariate, δ>0 indicates movement to areas with higher values, and δ=0 indicates no movement response. For example, to constrain a marine mammal’s movements so that it generally remains off land, the habitat covariate could be the shortest distance to water and δ a relatively large negative number (Fig. 3f). Similar to Eq. 6, centers of attraction can also be modeled using potential functions (with distance to a the covariate and δ<0). Other examples of habitat features that could be incorporated into potential functions include elevation, wind velocity, ocean currents, sea surface temperature, sea ice concentration, snow cover, fenced wildlife enclosures, and stream, trail, or road networks (Brillinger 2003, Hanks et al. 2011, Hooten et al. 2019*b*). The potential surface could also be based on the standard parametric form for RSFs (Manly et al. 2007) using an approximate Langevin diffusion movement model (Michelot et al. 2019*b*):

(10) $$\mu_{t}=\mu_{t‐1}+\frac{\sigma^{2}}{2}\sum_{k\;=\;1}^{K}D_{k}\nabla c_{k}\left(\mu_{t‐1}\right)+\varepsilon_{t},$$

where K is the number of habitat covariates included in the RSF, $c_{k}\left(\mu_{t‐1}\right)$ is the kth covariate evaluated at $\mu_{t‐1}$, $D_{k}=\delta_{k}I$, and $\varepsilon_{t}∼N\left(0,\sigma^{2}I\right)$. Potential functions have also been utilized in state‐switching models that account for physiological processes associated with decision making and movement in heterogeneous environments (Hooten et al. 2019*b*).

We can thus capture various features of animal movement through combinations of these basic building blocks using the general formula $\mu_{t}|\theta ∼N\left(\mu_{t}^{*},\sum \right)$, where $\mu_{t}^{*}=E\left(\mu_{t}\right)$ is the expected value for $\mu_{t}$ and θ is the vector of parameters embedded in $\mu_{t}^{*}$ and ∑. For example, $\mu_{t}^{*}=\mu_{t‐1}$ and $\theta =\left(\sigma \right)$ for Eq. 3, $\mu_{t}^{*}=\mu_{t‐1}+R\left(\mu_{t‐1}‐\mu_{t‐2}\right)$ and $\theta =\left(\gamma ,\beta ,\sigma \right)$ for Eq. 4, $\mu_{t}^{*}=\mu_{t‐1}+B\left(a‐\mu_{t‐1}\right)$ and $\theta =\left(\rho ,\sigma \right)$ for Eq. 6, and so forth. Although we have demonstrated these basic building blocks using positions in continuous space $\left(\mu_{t}\right)$, alternatively space can be discretized (Hooten et al. 2010, Hanks et al. 2015, Wilson et al. 2018) and/or other movement metrics such as velocity (Jonsen et al. 2005, Johnson et al. 2008, Hanks et al. 2011, Gurarie et al. 2017) or steps and turns (Franke et al. 2004, Morales et al. 2004, Langrock et al. 2012, McClintock et al. 2012, Parton and Blackwell 2017) can be used.

#### Limiting distributions

One movement model concept that is useful when transitioning from Lagrangian to Eulerian inference is the *limiting distribution* of the time series process (Norris 1998). The limiting distribution is defined as

$$\pi \left(\mu \right)=\underset{h\to \infty }{\text{lim}}\left[\mu_{t+h}|\mu_{t}\right].$$

That is, as we consider greater time gaps, the distribution of the location “forgets” the past and is not dependent on the location at time t. This allows Eulerian inference from individual movement models because if we assume that all N animals in M have been moving for a long time according to the same process model, then the limiting distribution can be interpreted as an animal density surface (Brillinger et al. 2012, Wilson et al. 2018).

Unfortunately, not all movement models have limiting distributions. Fewer still have closed form limiting distributions. The biased random walk (Eq. 6) is one that does:

(11) $$\pi_{\text{BRW}}\left(\mu \right)\equiv N\left(a,{\left(1‐{\left(1‐\rho \right)}^{2}\right)}^{‐1}\sum \right)$$

(Lütkepohl 2013). The standard SCR observation model can be thought of as a nonuniform thinning of this limiting distribution. In other words, the probability that an animal is captured depends on both the probability that it occurs at a location (described by the limiting distribution) and the probability that a trap occurs at (or near) that location. This implies that the limiting distribution is operational at small time gaps, which is unrealistic because it implies that an individual can be “everywhere at once” within its home range. Nonetheless, using the limiting distribution of a movement model as the basis of the SCR observation model can be a reasonable simplification of reality when sampling occasions are sufficiently long for an animal to move throughout its home range during an occasion, thereby diminishing temporal autocorrelation in the detection histories (Borchers et al. 2014). Note that the variance in Eq. 11 grows infinite as ρ→0 (i.e., as the model reverts to a simple random walk), hence the simple random walk has no limiting distribution.

Another movement model where an approximate limiting distribution can be provided is the Langevin diffusion RSF model (Eq. 10). As Δt→0, this model approaches a continuous‐time Langevin diffusion, which has a closed‐form limiting distribution (Roberts and Tweedie 1996, Michelot et al. 2019*b*):

$$\pi_{\mathrm{LD}}\left(\mu \right)=\frac{\exp\left(\sum_{k=1}^{K}\delta_{k}c_{k}\left(\mu \right)\right)}{\int_{M}\exp\left(\sum_{k=1}^{K}\delta_{k}c_{k}\left(z\right)\right)dz}.$$

This is the standard form of a RSF (Manly et al. 2007). Thus, if the movements are small enough relative to the timescale of the locations $\left(\Delta t\right)$, $\delta_{k}$ can be interpreted as a resource selection coefficient. Michelot et al. (2019*b*) provide a method for determining if Δt is sufficiently fine for this approximation to be acceptable.

#### Measurement error

An important complication with observations of animal locations is that they can be subject to measurement errors, such as those arising from GPS or Argos satellite telemetry data (Costa et al. 2010). Observed locations $\left(u_{t}\right)$ that are subject to measurement error are typically modeled hierarchically by conditioning on the true but unknown location $\left(\mu_{t}\right)$. For example,

(12) $$\left[u|\theta \right]=\int_{M}\overset{\text{measurement model}}{\overbrace{\left[u|\mu ,\theta \right]}}\underset{\text{movement model}}{\underbrace{\left[\mu |\theta \right]}}d\mu ,$$

where $\left[u_{t}|\mu_{t},\theta \right]\equiv N\left(\mu_{t},\sum_{u}\right)$, $\left[\mu_{t}|\theta \right]\equiv N\left(\mu_{t}^{*},\sum \right)$, θ is the vector of all observation and movement process parameters, and the measurement error covariance matrix $\left(\sum_{u}\right)$ is either informed by auxiliary data or estimated from the observations (Jonsen et al. 2005, Brost et al. 2015, McClintock et al. 2015). This represents just one of many ways these probability density functions, $\left[u|\mu ,\theta \right]$ and $\left[\mu |\theta \right]$, could be specified for joint modeling of the observation and movement processes. Another complication is that observations do not always occur at regular time intervals as assumed in the discrete‐time models described above. Although there are methods for accounting for temporally irregular observations in discrete‐time models (Jonsen et al. 2005, McClintock 2017), this, of course, is not an issue for continuous‐time formulations.

## Integrating Movement and Spatial Capture–Recapture

### Motivation

SCR has provided a new avenue for making inferences about population abundance and density, home range size, survival, recruitment, landscape connectivity, age structure, disease prevalence, species co‐occurrence, resource selection, and responses to disturbance or environmental change (Royle et al. 2013*b*). However, its implicit assumptions about animal movement and space use come at a cost. In standard SCR, the scale parameter $\left(\sigma_{\text{det}}\right)$ is only relevant to home range size when activity centers are static, the home range is symmetric, and when animal locations are statistically independent (Royle et al. 2016). In reality, this is clearly a crude and unrealistic description of space use by complex organisms responding to internal and external drivers (Fig. 1). These assumptions have also limited the use of SCR for nomadic populations, such as polar bears, with applications instead tending to focus on range resident (e.g., territorial) species. Although abundance (or density) point estimators have generally been shown to exhibit little bias with noncircular home ranges (Efford 2019, 2019) or dynamic activity centers (Royle et al. 2016), uncertainty is often underestimated (resulting in less than nominal confidence or credible interval coverage) and considerable bias is induced in the scale parameter as a measure of home range size (Royle et al. 2016). When space use within the home range is not random (e.g., resource selection), this can induce severe bias in abundance (or density) because of unmodeled heterogeneity in the detection process (Royle et al. 2013*c*). In open population models, movement can bias estimates of survival, density, and detection function parameters, as well as induce sensitivity to the specification of the state space (Gardner et al. 2018, Glennie et al. 2019). In addition, SCR models do not account for spatio‐temporal correlation in detections that can be induced by movement processes, which can result in overly precise estimates (Borchers et al. 2014). Thus although standard SCR can provide novel insights into spatial population dynamics, the existing framework does not properly account for complex animal movements and space use. Articles in the Special Feature investigate the consequences of ignoring complex movement in terms of statistical inference and, perhaps more importantly, in terms of the missed opportunity to empirically test ecological hypotheses relating movement processes to population structure and dynamics (Dunning et al. 1995, Morales et al. 2010, Royle et al. 2018), and offer analytical solutions.

Animal movement models can address questions related to behavior, activity budgets, physiology, home range size, resource selection, landscape connectivity, intra‐ and interspecific interactions, drivers of movement (e.g., environmental gradients, memory), individual‐level variation, community or ecosystem function (e.g., disease or nutrient transfer), and responses to disturbance or environmental change (Nathan et al. 2008, Cagnacci et al. 2010, Bauer and Hoye 2014, Hooten et al. 2017, Abrahms et al. 2019). However, although many sophisticated models of animal movement and space use exist, few of them are embedded within a framework that allows for the estimation of population density or other demographic parameters. Indeed, much like conventional (nonspatial) capture–recapture, applications of animal movement models typically lack a clearly defined population in both space and time. Furthermore, population‐level inferences about animal movement and space use are typically based on a relatively small (and nonrandom) sample of telemetered individuals (Hebblewhite and Haydon 2010, Hays et al. 2020), with no attempt to account for the process by which individuals were tagged or other potential sampling biases. For example, telemetry data are often produced through sampling efforts that focus on the most accessible locations (e.g., near roads or ports) where animals are thought to be present (i.e., convenience sampling; Anderson 2001). Similar in spirit to the detection process in capture–recapture, the process by which individuals are encountered, captured, and deployed with telemetry devices can often be a complex combination of population distribution, animal behavior (e.g., naïve vs. experienced individuals), study design (e.g., effort), logistical constraints, and practical considerations. A well‐defined population in space and time could not only help link movement to population dynamics, but could also account for potential sampling biases in telemetry or other types of location observation networks (Pedersen and Weng 2013).

The integration of movement and SCR models can help solve these deficiencies and establish a new framework for scaling individual‐level data to population‐level processes. Although we focus on the first step of connecting the individual to the population, our framework would allow potential links between intrinsic and extrinsic factors, individual movement and performance, population and community dynamics, and ecosystem function to be empirically tested (Fig. 1). For example, integrated models could be used to investigate the role of different movement behaviors (e.g., transience, dispersal, territoriality, group‐dynamic movements) in population dynamics (or vice versa), explain the mechanisms underlying spatial variation in abundance or density, connect resource selection with species distribution to test optimal foraging theory, or link individual activity budgets to survival and reproduction. In addition to this potential for new ecological insights, integrated models can reduce bias and properly account for uncertainty when standard SCR assumptions about animal movement are violated. By formally disentangling movement and detection parameters, integrated models could also provide a coherent framework for combining SCR and auxiliary location data (Tenan et al. 2017). However, although the potential benefits of integrated movement and SCR models should by now be readily apparent, they are likely to come at the cost of additional data and model complexity. There remain many conceptual, technical, and practical hurdles that must be overcome for their full potential to be realized.

### General framework

The integration of movement and SCR models is a natural extension when viewed from the perspective that animal location and SCR encounter history data arise from the same underlying movement process (Royle et al. 2013*c*). For example, SCR data can be viewed as a type of location data that is thinned via imperfect detection at spatially discrete detectors $\left(x_{j}\right)$. Put another way, location data produce direct observations of space usage, and SCR data result from a non‐uniform thinning of such data. Heuristically, a general conceptual framework for integrating animal movement processes into SCR models can be described as

(13) $$\left[y|\mu ,N,\theta \right]=\underset{\text{SCR model}}{\underbrace{\left[y|\mu ,\theta \right]}}\overset{\text{movement model}}{\overbrace{\left[\mu |\theta \right]}}\underset{\text{point process model}}{\underbrace{\left[\mu_{1},N|\theta \right]}},$$

where the SCR and movement model components are linked by the underlying position process $\left(\mu \right)$, which, as a latent variable, must be integrated over the support of the state space $\left(\mu \in M\right)$ during model fitting (see *Model fitting*). So instead of modeling static activity centers as in standard SCR (Eq. 1), we are now modeling individual movement trajectories through time (Fig. 2). Incorporation of an explicit movement process model therefore changes the standard SCR model in two key respects: (1) detection probability in the SCR component $\left[y|\mu ,\theta \right]$ is now modeled as a function of the location of a detector $\left(x_{j}\right)$ and the location of an animal at time t $\left(\mu_{t}\right)$; and (2) instead of activity centers, the spatial point process model component $\left[\mu_{1},N|\theta \right]$ now describes the density and distribution of initial locations for the animals at time t=1 $\left(\mu_{1}\right)$. For movement models that include a central tendency towards a static center of attraction (Eq. 6), then a point process model for spatial variation in the density of centers of attraction could be specified for a:

(14) $$\left[y|\mu ,a,N,\theta \right]=\underset{\text{SCR model}}{\underbrace{\left[y|\mu ,\theta \right]}}\overset{\text{movement model}}{\overbrace{\left[\mu |a,\theta \right]}}\underset{\text{point process model}}{\underbrace{\left[\mu_{1}|a,\theta \right]}}\overset{\text{point process model}}{\overbrace{\left[a,N|\theta \right]}}.$$

For inhomogeneously distributed centers of attraction, this would allow simultaneously testing of hypotheses that distinguish second‐order resource selection (i.e., individual home range selection within M) from third‐order selection (i.e., resource selection within a home range; for example, Johnson [1980], Royle et al. [2018]). This would also allow for the case where animals have a center of attraction, but the sampling period is shorter than the time it would take for an individual to explore its entire home range. In what follows, we for simplicity focus on standard SCR observations where the spatial encounter history data $\left(y\right)$ are the outcomes of Bernoulli trials across a fixed array of J traps (Borchers and Efford 2008, Royle et al. 2009). However, the framework could be easily modified to accommodate other observation distributions (binomial or Poisson; Royle et al. 2013*b*: Chapter 9) or search‐encounter data (Royle et al. 2013*b*: Chapter 15).

Although conceptually straightforward, several challenges arise when actually trying to formulate such models. First and foremost is the treatment of time. Continuous‐time formulations arguably provide the most natural framework for integrating instantaneous detection (e.g., camera trap) and movement process models, but most SCR and movement models are formulated in discrete time where each of the T sampling occasions corresponds to an interval (e.g., hourly, daily, weekly) from time t‐1 to time t. Clearly, an animal does not instantaneously move at time t from location $\mu_{t‐1}$ to $\mu_{t}$; nor is it detected exactly at time t‐1 or t. A continuous‐time movement model (or a discrete‐time approximation) could be combined with a discrete‐time SCR model, whereby the detection probability over the interval is handled similarly to search‐encounter SCR models (Efford 2011, Royle et al. 2011) but in “reverse” (i.e., the animal movement path is the search path and the detector is the animal location):

(15) $$p_{\mathrm{ijt}}=1‐\exp\left(‐\int_{t‐1}^{t}h\left(x_{j},\mu_{i\tau }\right)d\tau \right),$$

where $p_{\mathrm{ijt}}$ is the detection probability for individual i at trap j from time t‐1 to time t, $\mu_{i\tau }$ is the location of individual i at time τ, and $h\left(x_{j},\mu_{i\tau }\right)$ is the hazard to detection at time τ for trap j. The hazard would typically be a function of the distance from trap j to the location of the animal at time τ, for example, $\text{log}\left(h\left(x_{j},\mu_{i\tau }\right)\right)=\beta_{0}+\beta_{1}∥x_{j}‐\mu_{i\tau }∥$, where $\beta_{0}$ is the (log‐scale) baseline instantaneous detection rate at zero distance, $∥x_{j}‐\mu_{i\tau }∥$ is some distance metric, and $\beta_{1}$ the corresponding coefficient. Although Eq. 15 shares conceptual similarities with search‐encounter SCR detection functions, it poses a more challenging problem because the animal movement path is typically unknown (see *Challenges*).

For a SCR movement model formulated entirely in discrete time, both the instantaneous time of detection and the instantaneous location of the animal are ignored. We must therefore account for the fact that an animal can both move and be detected at any time between times t‐1 and t. One way to accomplish this is to model the SCR detection process as a function of the distance from the detector to the *expected* location of an animal $\left(s_{t}\right)$ from time t‐1 to time t. We can then specify a movement model for $s_{t}$ and formulate the detection process at trap j and time t as a function of the distance between $s_{t}$ and $x_{j}$:

(16) $$\left[y|s,N,\theta \right]=\underset{\text{SCR model}}{\underbrace{\left[y|s,\theta \right]}}\overset{\text{movement model}}{\overbrace{\left[s|\theta \right]}}\underset{\text{point process model}}{\underbrace{\left[s_{1},N|\theta \right]}},$$

where, for example,

(17) $$p_{\mathrm{ijt}}=p_{0}\exp\left(‐\frac{{∥x_{j}‐s_{\mathrm{it}}∥}^{2}}{2\sigma_{\text{det}}^{2}}\right)$$

is based on the kernel of a Gaussian probability density, $p_{0}$ is the baseline detection probability at distance zero, and $∥x_{j}‐s_{\mathrm{it}}∥$ is the Euclidean distance between the expected location for individual i from time t‐1 to t $\left(s_{\mathrm{it}}\right)$ and $x_{j}$. Royle et al. (2016) investigated precisely this model using a simple random walk (Eq. 3) for $\left[s|\theta \right]$ and a homogeneous binomial point process model for $\left[s_{1},N|\theta \right]$ to account for transients with nonstationary home ranges, but more realistic movement and point process models could be used.

As a more concrete example, suppose we have a population composed of resident and transient individuals, where residents utilize space according to the Langevin diffusion RSF approximation (Eq. 10) and transients follow a correlated random walk (Eq. 4): $\left[s_{\mathrm{it}}|m_{i},\theta \right]\equiv N\left(s_{\mathrm{it}}^{*},\sigma_{m_{i}}^{2}I\right)$, where

(18) $$s_{\mathrm{it}}^{*}=\left\{\begin{array}{cc} s_{i,t‐1}+\frac{\sigma_{1}^{2}}{2}\sum_{k=1}^{K}D_{k}\nabla c_{k}\left(s_{i,t‐1}\right) & \text{if}\;m_{i}=1\;\left(\text{resident}\right)\\ s_{i,t‐1}+R\left(s_{i,t‐1}‐s_{i,t‐2}\right) & \text{if}\;m_{i}=2\;\left(\text{transient}\right) \end{array}\right.,$$

and we might expect transients to exhibit greater degrees of dispersion than residents (i.e., $\sigma_{2}\;>\;\sigma_{1}$). If the movements are small relative to the timescale of locations, the expected population‐level utilization distribution for residents could be approximated by the standard RSF point process model:

(19) $$\pi \left(s\right)=\frac{\exp\left(\sum_{k=1}^{K}\delta_{k}c_{k}\left(s\right)\right)}{\int_{M}\exp\left(\sum_{k=1}^{K}\delta_{k}c_{k}\left(z\right)\right)dz},$$

where $c_{k}\left(s\right)$ is the value of the kth habitat covariate at location s. We could then have different point process models describing the distribution of initial locations based on whether an individual is a resident or a transient:

(20) $$\left[s_{i1}|m_{i},\theta \right]=\left\{\begin{array}{cc} \pi \left(s_{i1}\right) & \text{if}\;m_{i}=1\;\left(\text{resident}\right)\\ \text{Uniform}\left(M\right) & \text{if}\;m_{i}=2\;\left(\text{transient}\right) \end{array}\right..$$

This formulation has some useful properties in that it can account for nonstationary and nonrandom space use while ensuring that the resident movement model scales to the population‐level utilization distribution that is used for the point process model of the initial locations. As illustrated by the basic building blocks in *Movement modeling*, alternative or additional movement processes could be incorporated into these components based on the life history of the specific species under consideration (e.g., territorial or group‐dynamic behavior). For example, the Langevin diffusion RSF model (Eq. 18) could be extended to range resident movement behavior, where each individual has a center of attraction as in Eq. 14 (see Appendix S1). However, although we believe these illustrative integrated SCR movement models are promising, we note that they have yet to be implemented and in practice may pose significant challenges (see *Challenges*).

We have focused on demonstrating how more realistic movement models can be integrated with SCR models, but the SCR component does not need to be limited to standard closed population models (Borchers and Efford 2008, Royle et al. 2009). SCR models can describe open populations, such as those that utilize the robust design (Ergon and Gardner 2014) or Jolly‐Seber formulations (Glennie et al. 2019, Efford and Schofield 2020), to distinguish the roles of mortality, space use, temporary emigration, transience, and dispersal in population dynamics. Multistate SCR models could also be used to investigate other drivers of population structure and dynamics, such as sex or age composition, disease status, or reproductive trade‐offs (Nichols and Kendall 1995, Lebreton et al. 2009).

An obvious challenge to the integration of movement and SCR models is the amount of movement information contained in SCR data. SCR data sets are often sparse, which may make drawing inferences about movement difficult. Auxiliary location information (e.g., from animal‐borne telemetry or opportunistic data) can provide more detail about movement and space use as the extent of these data is not necessarily limited by a fixed trapping array and many more locations can potentially be accumulated than through capture alone. If auxiliary location information $\left(u\right)$ is available, we can include an additional component to account for any location measurement error:

(21) $$\left[y,u|\mu ,N,\theta \right]=\underset{\text{SCR model}}{\underbrace{\left[y|\mu ,\theta \right]}}\overset{\text{measurement model}}{\overbrace{\left[u|\mu ,\theta \right]}}\underset{\text{movement model}}{\underbrace{\left[\mu |\theta \right]}}\overset{\text{point process model}}{\overbrace{\left[\mu_{1},N|\theta \right]}}.$$

However, combining SCR detection and auxiliary location data in a coherent fashion will likely pose some additional technical challenges (Royle et al. 2013*b*, Tenan et al. 2017; see *Looking Forward: Where Are We Going?*), and this is an area where continuous‐time formulations may be particularly appealing (Borchers et al. 2014). By again conditioning the SCR component on s instead of μ, one of the simplest ways this could be accomplished in discrete time (Eq. 16) follows Sollmann et al. (2013), where some segment of the population has been deployed with telemetry tags. Assuming any telemetry locations observed between times t‐1 and t (i.e., $u_{i\tau }$ for t‐1≤τ≤t) are subject to measurement error, these could be modeled as $\left[u_{i\tau }|\mu_{i\tau },\theta \right]\equiv N\left(\mu_{i\tau },\sum_{u}\right)$ and, using an additional model component, the auxiliary true locations as $\left[\mu_{i\tau }|s_{\mathrm{it}},\theta \right]\equiv N\left(s_{\mathrm{it}},\sigma_{\text{det}}^{2}I\right)$. However, this highlights a key disadvantage of formulating these models in discrete time using a movement model for s instead of μ; ignoring the instantaneous times of detection and the instantaneous locations of individuals introduces ambiguity in the interpretation of $\sigma_{\text{det}}$ and the movement parameters as they relate to the underlying (true) movement process $\left[\mu |\theta \right]$. Although integrated SCR movement models in discrete time are an important and accessible first step, we anticipate that continuous‐time formulations that condition on the true position process will begin to materialize in the near future.

## Looking Forward: Where Are We Going?

### Challenges

The integration of movement and SCR models has been identified as a promising research area for years, but progress has thus far been limited. This is likely attributable to the considerable challenges these extensions will pose. Formulating SCR models with explicit movement processes will likely require expertise from both fields, but provides an exciting opportunity for increased collaboration between ecologists studying animal movement and those studying population processes. As integrated models become more complex, they have the potential to push the limits of spatial detection history data and may require the integration of multiple data sources to inform the underlying movement and population processes. Even with sufficient data and/or prior information, the models are also likely to be very challenging to fit and may require additional statistical expertise to develop suitable model‐fitting algorithms. Below we detail some of the key challenges that lie ahead.

#### Data requirements

Little is currently known about optimal study design and data requirements for integrated SCR movement models. In standard SCR, precision of density estimates depends on the number of individuals captured (n) and the number of spatial recaptures. A trade‐off exists in efforts to maximize both quantities with a fixed number of traps. The number of individuals captured can be maximized by distributing traps over a large area, but sparse trap configurations will decrease the frequency of spatial recaptures. Conversely, dense clusters of traps can improve the spatial recapture rate, but at the expense of n. Effective SCR study designs therefore achieve a balance between these two design objectives. For specific applications with associated logistical constraints, an optimal design can be found using either simulation studies (Efford 2011, Marques et al. 2011, Sollmann et al. 2012, Sun et al. 2014) or by optimizing design criteria (Royle et al. 2013*b*, Williams et al. 2018, Efford and Boulanger 2019, Durbach et al. 2020, Dupont et al. 2021). Many of these studies have indicated that traps should be spaced by approximately 1–2σ units. However, it is unknown if these general recommendations will hold in the context of integrated SCR movement models. It seems likely that the spatial recapture rate would need to be higher to estimate the parameters of the movement model, and thus clustered designs may be more useful than in standard SCR, but similar simulation studies and optimization methods will be required to understand what constitutes an optimal design when using integrated SCR movement models.

Relatively simple movement models will require few additional parameters, and Royle et al. (2013*c*), Royle et al. (2013*a*), and Royle et al. (2016) have already demonstrated that SCR data alone can be sufficient for inferences about animal movement, space use, and resource selection. However, these have only considered models with constant density throughout the survey region, and sparse SCR data may not support complex animal movement and density processes without additional information (Gardner et al. 2018). Telemetry and opportunistic location data have been widely used in conjunction with capture–recapture data (White and Shenk 2001, Royle et al. 2013*c*, Sollmann et al. 2013, Tenan et al. 2017, Linden et al. 2018) and could be integrated as additional likelihood components (as described in *Integrating Movement and Spatial Capture–Recapture*) or as the basis for informative prior distributions in Bayesian analyses. But integrating different data sources will present additional challenges, such as lining up the temporal resolution of concurrently collected SCR and telemetry data (see *Spatio‐temporal formulation*) or telemetered individuals not always being identified as such when detected by a trap (see *Future directions*). Concurrent SCR and telemetry projects will often be expensive, and many wildlife studies simply cannot allocate adequate resources to both. Although opportunistic (e.g., citizen science) data can often be less expensive, use of such records will often necessitate additional modeling to account and correct for sampling biases.

The last point about sampling biases applies more broadly; when integrating different sources of data, there is a larger issue of data consistency (Tenan et al. 2017). Uncorrected sampling biases in any data collection protocol or an incoherent linkage of shared model parameters could result in unreliable inferences. Although standard SCR models account for detection bias in spatial encounter history data, sampling biases in the telemetry tagging process are typically ignored in animal movement studies. Integrating these different data sources in a SCR movement model will be problematic if they are not representative samples from the same underlying population of interest. Fortunately, there are ways in which such inconsistencies can be detected. For instance, if utilization distributions from telemetry studies do not match up with spatial density surfaces produced from simple SCR models, this would strongly indicate that the telemetry sample is not representative of the population.

Much like in capture–mark–resight studies (Matechou et al. 2013, Efford and Hunter 2018) or search‐encounter SCR models (Royle et al. 2013*b*: Chapter 15), there may be ways to account for the telemetry tagging process (e.g., search–encounter live trapping) when it differs from the SCR detection process (e.g., camera trapping). However, this may require changes in standard tag deployment protocols to account for tagging probability and thereby correct for any such sampling biases. For example, it may be necessary to record the entire search path, all individual encounters (whether pursued for capture or not), and any unsuccessful capture attempts for inclusion in an observation model describing the tagging process. We also note that SCR models incorporating auxiliary location data typically assume independence of these data sources (Royle et al. 2013*c*), but, by coherently linking these data to the same underlying position process, integrated SCR movement models provide a natural framework to account for spatio‐temporal dependencies in the data.

#### Spatio‐temporal formulation

**Fig. 4:**
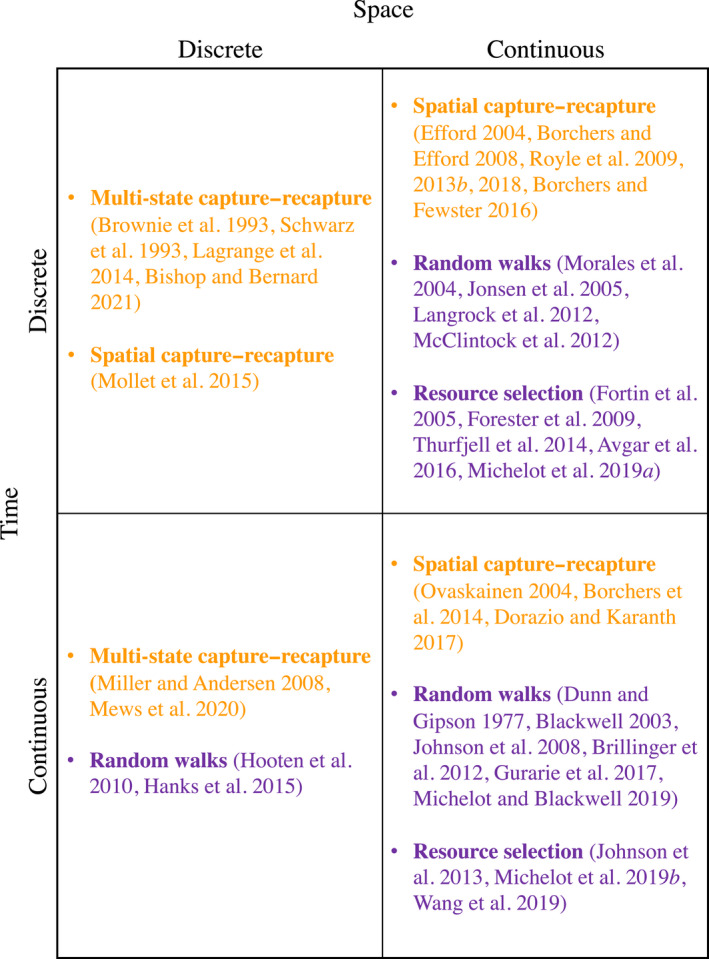
Examples of different formulations of capture–recapture models for spatially referenced encounter history data (orange) and animal movement models typically used for telemetry data (purple), organized by spatial and temporal support (discrete or continuous).

Although we have largely focused on discrete‐time, continuous‐space models for ease of exposition and because these are most common in the literature, under certain study designs alternative spatio‐temporal formulations may have distinct advantages for integrated SCR movement modeling. In particular, we have only described models for one quadrant of the possible state space (Fig. 4). In practice, we imagine that choices of spatial and temporal support will be guided by characteristics of the sampling process, as well as the relative simplicity of modeling.

The feasibility of continuous‐time formulations for the SCR component (bottom right quadrant of Fig. 4) is largely driven by the study design and whether or not it results in instantaneous detection times of individuals. For example, camera‐trap or search‐encounter data can both provide instantaneous detection times that are amenable to continuous‐time formulations (Borchers et al. 2014), whereas traditional live traps that hold an animal until release, such as commonly used for small mammals, typically do not provide instantaneous detection times and therefore necessitate discrete‐time formulations. When instantaneous detection times are available, Borchers et al. (2014) identified several important advantages of continuous‐time models that can utilize this additional information, including the potential for continuous‐time SCR models to be combined with telemetry‐based movement models.

For the movement model component, the choice of discrete or continuous time usually represents a trade‐off (McClintock et al. 2014). Animals clearly move in continuous time, but their movement is typically observed in discrete intervals. Discrete time has likely been more popular in animal movement modeling because it can often be more intuitive and easier to implement than continuous time. For example, some of the most popular discrete‐time models for animal movement behavior or resource selection (Franke et al. 2004, Morales et al. 2004, Fortin et al. 2005) are relatively easy to implement with user‐friendly software (Michelot et al. 2016, McClintock and Michelot 2018, Signer et al. 2019), whereas their continuous‐time analogues require custom model‐fitting algorithms that are very computationally demanding (Parton and Blackwell 2017, Michelot and Blackwell 2019, Wang et al. 2019). However, continuous‐time formulations have desirable qualities in that they do not require regular time steps over discrete intervals and the movement parameters are scale‐invariant to the time step. Thus, although continuous‐time models can sometimes be less intuitive and come at greater computational cost, they may be better suited for SCR data collected in continuous time (e.g., camera traps), especially when combined with irregularly observed telemetry or opportunistic data. Unlike the discrete‐time formulation in Eq. 16 that models the movement of the *expected* location of an animal over an interval of time $\left(s\right)$, a continuous‐time formulation would naturally link instantaneous detection (and any auxiliary location) data to the true underlying position process $\left(\mu \right)$ as in Eq. 13. However, although continuous time is conceptually pleasing and mathematically elegant, the practical trade‐offs between discrete and continuous time are little understood in this context and warrant further investigation.

Both SCR and movement models tend to be formulated in continuous space, but they do not necessarily need to be. It can often be convenient to use a discretization of space for incorporating suitable habitat masks or other covariates in raster form (Hijmans 2020). Indeed, frequentist SCR models (and Bayesian models based on a semicomplete data likelihood) that are formulated in continuous space typically rely on a discretization of space to approximate the integral over the activity centers (Borchers and Efford 2008, King et al. 2016, Glennie et al. 2019; see *Model fitting*). As the pixel size of the discretization approaches zero, the approximation becomes equivalent to continuous space. Movement models can also be formulated in discrete space as a means for making inferences about space use and resource selection more computationally tractable (Hanks et al. 2015). Instead of focusing on the microscopic rules of movement in continuous space (e.g., steps and turns) these models concentrate on the directional drivers of movement between adjacent pixels. Little is currently known about the potential advantages and disadvantages of formulating integrated SCR movement models in continuous or discrete space, but these will likely depend on study objectives and computational convenience.

#### State space

Whether formulated in continuous or discrete space, an important complication of incorporating explicit movement processes into SCR models is that animals could potentially leave the state space $\left(M\right)$. For example, a (nonstationary) simple or correlated random walk movement model would result in an unreasonable decrease in density over time (Royle et al. 2013*b*: Chapter 16). Even a (stationary) movement model that includes a center of attraction $\left(a\right)$ would allow an individual to leave the state space if a is near the boundary. There are several obvious approaches to dealing with this issue, but the practical utility of each remains poorly understood. The simplest option is to simply forbid movements outside of an arbitrary state space using a boundary mask (in discrete space) or truncation (in continuous space), but this could induce undesirable edge effects on the movement properties of individuals that use space near the boundary of the state space. An alternative would be to use a potential function (Eq. 9) to pull animals away from the boundary, which could provide a more elegant and coherent means for handling edge effects, but could still result in unrealistic movement near an arbitrary boundary. Another option that will be essential for animals that move long distances (e.g., polar bears) is expanding the state space to a very large area such that individuals using space outside this area have a negligible chance of being detected, but very large state spaces will likely come at significant computational cost (see *Model fitting*). A more ecologically driven approach would be to define and enforce state space boundaries based on a suitable habitat mask that reflects the underlying “true” boundaries of movement, but this could again result in extremely large state spaces and additional parameters to be estimated. No matter which approach is used, integrated SCR movement models may require larger state spaces and additional parameters to keep animals within the state space, and, when compared to standard SCR, this will likely come at the cost of increased computational complexity when fitting these models.

#### Model fitting

Despite their additional complexity, formulating integrated SCR movement model likelihoods is arguably the “easy” part. Actually fitting such models to data will likely present many technical and computational challenges. As with standard SCR, the root of these model‐fitting challenges is removing the latent locations from the conditional likelihood (Eq. 13) by integrating over the support of the state space. However, the latent locations now pertain to entire movement paths instead of static activity centers. This means that for a discrete‐time model consisting of T occasions, there are now NT latent variables that must be accounted for. This multidimensional integral will typically not have a closed‐form solution and must be approximated using numerical methods, such as quadrature (Borchers and Efford 2008) or Monte Carlo integration (Royle et al. 2009, 2016). Discrete approximations using hidden Markov model (HMM) algorithms are likely to be useful for this purpose (Glennie et al. 2019, Efford and Schofield 2020). In essence, this approach treats each discrete pixel in M as a “state” and the state transitions correspond to the probability of moving from pixel i at time t‐1 to pixel j at time t. Given an initial pixel location at time t=1, the forward algorithm (also known as filtering) can then be used to marginalize over all possible trajectories within the state space, with each trajectory weighted by its likelihood given the spatial detection history (and any auxiliary location) data. For the large state spaces that integrated SCR movement models are likely to require, HMMs in conjunction with sparse matrix methods are particularly promising when animal movements are limited to neighboring pixels (Thygesen et al. 2009, Pedersen et al. 2011, Glennie et al. 2019). This is a biologically reasonable assumption for many species, and an appropriate pixel neighborhood could be derived from the underlying movement model, the expected rate of dispersion, habitat features, cost functions, or other factors.

Depending on the approach used for model fitting, in practice the dimension of the integral over space will likely be (much) less than NT (Borchers and Efford 2008) or (much) greater than NT (Royle et al. 2009). Bayesian Markov chain Monte Carlo (MCMC) approaches using data‐augmented complete data likelihoods and a binomial model for N (Royle et al. 2007, 2013*b*: Chapter 5) will require an integral of dimension *MT* (*M* ≫ *N*), and, for sparse data, slow mixing chains, and/or very large populations, large M may be computationally prohibitive (King et al. 2016). The dimension of the integral can be reduced substantially to nT using a semi‐complete data likelihood approach in either a frequentist (Borchers and Efford 2008) or Bayesian (King et al. 2016) setting, where n is the number of animals that were detected over the course of the study. When using this approach to condition the SCR model component on the observed individuals, abundance (or density) estimation requires an overall probability of detection $\left(p^{*}\right)$ for the N‐n unobserved individuals:

$$p^{*}=1‐\int_{M}\left[y=0|\theta ,\mu \right]\left[\mu |\theta \right]d\mu ,$$

where y=0 indicates spatial encounter history data consisting entirely of zeros (i.e., individuals that were never detected). Although this is relatively straightforward in standard SCR models with static activity centers (Borchers and Efford 2008, King et al. 2016), it will be more computationally expensive for integrated SCR movement models, and perhaps prohibitively so for very large state spaces. Whether used for frequentist or Bayesian inference, this is another area where HMMs and sparse matrix methods may be able to provide substantial gains in computational feasibility and efficiency. Other strategies that can be useful for large data sets and/or complex hierarchical structures include multistage model fitting techniques such as multiple imputation (McClintock 2017, Scharf et al. 2017) and recursive Bayesian computing (Hooten et al. 2019*a*, 2020, McCaslin et al. 2020).

Movement models formulated in continuous time also involve integrals, but, instead of being over space, the integration is with respect to time. Thus integrated SCR movement models formulated in continuous time will likely require multidimensional integrals over both space *and* time. As demonstrated in Eq. 15, when a discrete‐time SCR model (e.g., for live trapping data) is combined with a continuous‐time movement model, integration over time would be required not only to evaluate the movement model but also to calculate the (discrete‐time) detection probability from time t‐1 to time t. Evaluating the integral over time will also typically require numerical methods, such as discrete‐time approximations (Euler‐Maruyama method; Kloeden and Platen 1992, Hooten et al. 2019*b*, Michelot et al. 2019*b*) or filtering (Kalman filter; Johnson et al. 2008). Continuous‐time formulations may provide many nice properties, but they will also bring an additional level of computational complexity to model fitting.

### Future directions

There remains much work to be done, but the integration of movement and SCR modeling will be worth the effort. There are many other areas of potential development, and integrated SCR movement models could be expanded to include any of the many recent extensions from either field (Royle et al. 2013*b*, Borchers and Fewster 2016, Hooten et al. 2017, Patterson et al. 2017), including open population models (Gardner et al. 2010, Schaub and Royle 2014, Glennie et al. 2019, Efford and Schofield 2020), multistate models (Morales et al. 2004, Lebreton et al. 2009), physiological processes (Hooten et al. 2019*b*), group‐dynamic movements (Langrock et al. 2014), landscape connectivity (Royle et al. 2018), and misclassification or partial identity models (Link et al. 2010, Bonner and Holmdberg 2013, McClintock et al. 2013, Augustine et al. 2018, Maronde et al. 2020).

Much like standard SCR, we expect initial developments and applications will focus on closed population models, such as those in the Special Feature. However, populations of course have more structure than their overall abundance and distribution. Age, sex, breeding status, fitness, and behavioral or genetic heterogeneity all have an impact on the composition and development of a population (Seber and Schofield 2019). Combining these individual‐level attributes in multi‐state open population SCR models that explicitly model survival, recruitment, and patterns of movement or dispersal as a function of state (e.g., sex or age class) could provide a very rich framework for investigating how decision making and movement in heterogeneous environments affect population vital rates, dynamics, and distribution. These models are likely to be more data hungry, and when combining spatial encounter history data, auxiliary location data (e.g., telemetry), and other sources of data that can be informative about population dynamics (e.g., unmarked counts), integrated movement and open population SCR models can be viewed as an extension of the broader class of integrated population dynamics models (Schaub and Abadi 2011, Chandler and Clark 2014, Zhao 2020) to accommodate more realistic animal movement and space use.

Continued technological advancements will help make these models more feasible. Improvements in remote sensing and biotelemetry technology are likely to make data collection easier and less expensive, while faster and more powerful computers will make the fitting of complicated models more computationally tractable. Newer, faster, and more efficient algorithms may also be needed to handle more complex models, big data sets, or very large state spaces. In the meantime, we hope our review and synthesis of SCR movement models will motivate additional research beyond the Special Feature. In particular, we hope it will inspire novel empirical testing of ecological theory, provide ideas for quantitative ecologists and graduate students seeking research projects, and promote collaboration among ecologists, statisticians, and computer scientists in tackling the challenging and exciting opportunities that lie ahead.

## Supporting information

Appendix S1

## References

[ecy3473-bib-0001] Abrahms, B. , E. L. Hazen , E. O. Aikens , M. S. Savoca , J. A. Goldbogen , S. J. Bograd , M. G. Jacox , L. M. Irvine , D. M. Palacios , and B. R. Mate . 2019. Memory and resource tracking drive blue whale migrations. Proceedings of the National Academy of Sciences of the United States of America 116:5582–5587. 30804188 10.1073/pnas.1819031116 PMC6431148

[ecy3473-bib-0002] Anderson, D. R. 2001. The need to get the basics right in wildlife field studies. Wildlife Society Bulletin (1973–2006) 29:1294–1297.

[ecy3473-bib-0003] Augustine, B. C. , et al. 2018. Spatial capture–recapture with partial identity: An application to camera traps. Annals of Applied Statistics 12:67–95.

[ecy3473-bib-0004] Avgar, T. , J. R. Potts , M. A. Lewis , and M. S. Boyce . 2016. Integrated step selection analysis: bridging the gap between resource selection and animal movement. Methods in Ecology and Evolution 7:619–630.

[ecy3473-bib-0005] Bauer, S. , and B. J. Hoye . 2014. Migratory animals couple biodiversity and ecosystem functioning worldwide. Science 344:1242552. 24700862 10.1126/science.1242552

[ecy3473-bib-0006] Bishop, M. A. , and J. W. Bernard . 2021. An empirical Bayesian approach to incorporate directional movement information from a forage fish into the Arnason‐Schwarz mark–recapture model. Movement Ecology 9:8. 33627189 10.1186/s40462-021-00241-1 PMC7905629

[ecy3473-bib-0007] Blackwell, P. G. 2003. Bayesian inference for Markov processes with diffusion and discrete components. Biometrika 90:613–627.

[ecy3473-bib-0008] Bonner, S. J. , and J. Holmberg . 2013. Mark–recapture with multiple, non‐invasive marks. Biometrics 69:766–775. 23845216 10.1111/biom.12045

[ecy3473-bib-0009] Borchers, D. , G. Distiller , R. Foster , B. Harmsen , and L. Milazzo . 2014. Continuous‐time spatially explicit capture–recapture models, with an application to a jaguar camera‐trap survey. Methods in Ecology and Evolution 5:656–665.

[ecy3473-bib-0010] Borchers, D. L. , and M. Efford . 2008. Spatially explicit maximum likelihood methods for capture–recapture studies. Biometrics 64:377–385. 17970815 10.1111/j.1541-0420.2007.00927.x

[ecy3473-bib-0011] Borchers, D. , and R. Fewster . 2016. Spatial capture–recapture models. Statistical Science 31:219–232.

[ecy3473-bib-0012] Brillinger, D. R. 2003. Simulating constrained animal motion using stochastic differential equations. Lecture Notes–Monograph Series 41:35–48.

[ecy3473-bib-0013] Brillinger, D. R. , H. K. Preisler , A. A. Ager , and J. Kie . 2012. The use of potential functions in modelling animal movement. Pages 385–409 *in* Selected works of David Brillinger. Springer, New York, New York, USA.

[ecy3473-bib-0014] Brost, B. M. , M. B. Hooten , E. M. Hanks , and R. J. Small . 2015. Animal movement constraints improve resource selection inference in the presence of telemetry error. Ecology 96:2590–2597. 26649380 10.1890/15-0472.1

[ecy3473-bib-0015] Brownie, C. , J. E. Hines , J. D. Nichols , K. H. Pollock , and J. B. Hestbeck . 1993. Capture–recapture studies for multiple strata including non‐Markovian transitions. Biometrics 49:1173–1187.

[ecy3473-bib-0016] Byrne, M. E. , J. Clint McCoy , J. W. Hinton , M. J. Chamberlain , and B. A. Collier . 2014. Using dynamic Brownian bridge movement modelling to measure temporal patterns of habitat selection. Journal of Animal Ecology 83:1234–1243. 24460723 10.1111/1365-2656.12205

[ecy3473-bib-0017] Cagnacci, F. , L. Boitani , R. A. Powell , and M. S. Boyce . 2010. Animal ecology meets GPS‐based radiotelemetry: a perfect storm of opportunities and challenges. Philosophical Transactions of the Royal Society B 365:2157–2162. 10.1098/rstb.2010.0107 PMC2894970 20566493

[ecy3473-bib-0018] Calabrese, J. M. , C. H. Fleming , and E. Gurarie . 2016. ctmm: An R package for analyzing animal relocation data as a continuous‐time stochastic process. Methods in Ecology and Evolution 7:1124–1132.

[ecy3473-bib-0019] Carter, M. I. , B. T. McClintock , C. B. Embling , K. A. Bennett , D. Thompson , and D. J. Russell . 2020. From pup to predator; generalized hidden Markov models reveal rapid development of movement strategies in a naïve long‐lived vertebrate. Oikos 129:630–642.

[ecy3473-bib-0020] Chandler, R. B. , and J. D. Clark . 2014. Spatially explicit integrated population models. Methods in Ecology and Evolution 5:1351–1360.

[ecy3473-bib-0021] Chandler, R. B. , and J. A. Royle . 2013. Spatially explicit models for inference about density in unmarked or partially marked populations. Annals of Applied Statistics 7:936–954.

[ecy3473-bib-0022] Cooke, S. J. , S. G. Hinch , M. Wikelski , R. D. Andrews , L. J. Kuchel , T. G. Wolcott , and P. J. Butler . 2004. Biotelemetry: a mechanistic approach to ecology. Trends in Ecology & Evolution 19:334–343. 16701280 10.1016/j.tree.2004.04.003

[ecy3473-bib-0023] Costa, D. P. , et al. 2010. Accuracy of ARGOS locations of pinnipeds at‐sea estimated using Fastloc GPS. PLoS One 5:e8677. 20090942 10.1371/journal.pone.0008677 PMC2806907

[ecy3473-bib-0024] Dawber, P. G. 1987. Vectors and vector operators. CRC Press, Boca Raton, Florida, USA.

[ecy3473-bib-0025] Dickson, B. G. , et al. 2019. Circuit‐theory applications to connectivity science and conservation. Conservation Biology 33:239–249. 30311266 10.1111/cobi.13230 PMC6727660

[ecy3473-bib-0026] Distiller, G. B. , D. L. Borchers , R. J. Foster , and B. J. Harmsen . 2020. Using continuous‐time spatial capture–recapture models to make inference about animal activity patterns. Ecology and Evolution 10:11826–11837. 10.1002/ece3.6822 PMC7593165 33145005

[ecy3473-bib-0027] Dorazio, R. M. , and K. U. Karanth . 2017. A hierarchical model for estimating the spatial distribution and abundance of animals detected by continuous‐time recorders. PLoS One 12:e0176966. 28520796 10.1371/journal.pone.0176966 PMC5435310

[ecy3473-bib-0028] Dunn, J. E. , and P. S. Gipson . 1977. Analysis of radio telemetry data in studies of home range. Biometrics 33:85–101.

[ecy3473-bib-0029] Dunning, J. B. , D. J. Stewart , B. J. Danielson , B. R. Noon , T. L. Root , R. H. Lamberson , and E. E. Stevens . 1995. Spatially explicit population models: current forms and future uses. Ecological Applications 5:3–11.

[ecy3473-bib-0030] Dupont, G. , J. A. Royle , M. A. Nawaz , and C. Sutherland . 2021. Optimal sampling design for spatial capture–recapture. Ecology 102:e03262. 33244753 10.1002/ecy.3262

[ecy3473-bib-0031] Durbach, I. , D. Borchers , C. Sutherland , and K. Sharma . 2020. Fast, flexible alternatives to regular grid designs for spatial capture–recapture. Methods in Ecology and Evolution 12(2):298–310.

[ecy3473-bib-0032] Efford, M. 2004. Density estimation in live‐trapping studies. Oikos 106:598–610.

[ecy3473-bib-0033] Efford, M. G. 2011. Estimation of population density by spatially explicit capture–recapture analysis of data from area searches. Ecology 92:2202–2207. 22352159 10.1890/11-0332.1

[ecy3473-bib-0034] Efford, M. 2019. Non‐circular home ranges and the estimation of population density. Ecology 100:e02580. 30601582 10.1002/ecy.2580

[ecy3473-bib-0035] Efford, M. G. , and J. Boulanger . 2019. Fast evaluation of study designs for spatially explicit capture–recapture. Methods in Ecology and Evolution 10:1529–1535.

[ecy3473-bib-0036] Efford, M. G. , D. K. Dawson , and D. L. Borchers . 2009. Population density estimated from locations of individuals on a passive detector array. Ecology 90:2676–2682. 19886477 10.1890/08-1735.1

[ecy3473-bib-0037] Efford, M. G. , and C. M. Hunter . 2018. Spatial capture–mark–resight estimation of animal population density. Biometrics 74:411–420. 28834536 10.1111/biom.12766

[ecy3473-bib-0038] Efford, M. G. , and M. R. Schofield . 2020. A spatial open‐population capture–recapture model. Biometrics 76:392–402. 31517386 10.1111/biom.13150

[ecy3473-bib-0039] Ergon, T. , and B. Gardner . 2014. Separating mortality and emigration: modelling space use, dispersal and survival with robust‐design spatial capture–recapture data. Methods in Ecology and Evolution 5:1327–1336.

[ecy3473-bib-0040] Forester, J. D. , H. K. Im , and P. J. Rathouz . 2009. Accounting for animal movement in estimation of resource selection functions: sampling and data analysis. Ecology 90:3554–3565. 20120822 10.1890/08-0874.1

[ecy3473-bib-0041] Fortin, D. , H. L. Beyer , M. S. Boyce , D. W. Smith , T. Duchesne , and J. S. Mao . 2005. Wolves influence elk movements: behavior shapes a trophic cascade in Yellowstone National Park. Ecology 86:1320–1330.

[ecy3473-bib-0042] Franke, A. , T. Caelli , and R. J. Hudson . 2004. Analysis of movements and behavior of caribou (*Rangifer tarandus*) using hidden Markov models. Ecological Modelling 173:259–270.

[ecy3473-bib-0043] Gao, J. 2002. Integration of GPS with remote sensing and GIS: reality and prospect. Photogrammetric Engineering and Remote Sensing 68:447–454.

[ecy3473-bib-0044] Gardner, B. , J. Reppucci , M. Lucherini , and J. A. Royle . 2010. Spatially explicit inference for open populations: estimating demographic parameters from camera‐trap studies. Ecology 91:3376–3383. 21141198 10.1890/09-0804.1

[ecy3473-bib-0045] Gardner, B. , J. A. Royle , and M. T. Wegan . 2009. Hierarchical models for estimating density from DNA mark–recapture studies. Ecology 90:1106–1115. 19449704 10.1890/07-2112.1

[ecy3473-bib-0046] Gardner, B. , R. Sollmann , N. S. Kumar , D. Jathanna , and K. U. Karanth . 2018. State space and movement specification in open population spatial capture–recapture models. Ecology and Evolution 8:10336–10344. 30397470 10.1002/ece3.4509 PMC6206188

[ecy3473-bib-0047] Glennie, R. , D. L. Borchers , M. Murchie , B. J. Harmsen , and R. J. Foster . 2019. Open population maximum likelihood spatial capture–recapture. Biometrics 75:1345–1355. 31045249 10.1111/biom.13078

[ecy3473-bib-0048] Grecian, W. J. , J. V. Lane , T. Michelot , H. M. Wade , and K. C. Hamer . 2018. Understanding the ontogeny of foraging behaviour: insights from combining marine predator bio‐logging with satellite‐derived oceanography in hidden Markov models. Journal of the Royal Society Interface 15:20180084. 29875281 10.1098/rsif.2018.0084 PMC6030624

[ecy3473-bib-0049] Gurarie, E. , C. H. Fleming , W. F. Fagan , K. L. Laidre , J. Hernández‐Pliego , and O. Ovaskainen 2017. Correlated velocity models as a fundamental unit of animal movement: synthesis and applications. Movement Ecology 5:13. 28496983 10.1186/s40462-017-0103-3 PMC5424322

[ecy3473-bib-0050] Hanks, E. M. , M. B. Hooten, and M. W. Alldredge. 2015. Continuous‐time discrete‐space models for animal movement. Annals of Applied Statistics 9:145–165.

[ecy3473-bib-0051] Hanks, E. M. , M. B. Hooten , D. S. Johnson , and J. T. Sterling . 2011. Velocity‐based movement modeling for individual and population level inference. PLoS One 6:e22795. 21931584 10.1371/journal.pone.0022795 PMC3154913

[ecy3473-bib-0052] Hays, G. C. , A. Rattray , and N. Esteban . 2020. Addressing tagging location bias to assess space use by marine animals. Journal of Applied Ecology. 10.1111/1365-2664.13720

[ecy3473-bib-0053] Hebblewhite, M. , and D. T. Haydon . 2010. Distinguishing technology from biology: a critical review of the use of GPS telemetry data in ecology. Philosophical Transactions of the Royal Society B 365:2303–2312. 10.1098/rstb.2010.0087 PMC2894965 20566506

[ecy3473-bib-0054] Hijmans, R. J. 2020. raster: Geographic Data Analysis and Modeling. R package version 3.3‐13. https://CRAN.R‐project.org/package=raster

[ecy3473-bib-0055] Hooten, M. B. , D. S. Johnson , and B. M. Brost . 2019a. Making recursive Bayesian inference accessible. American Statistician 75(2):185–194.

[ecy3473-bib-0056] Hooten, M. B. , D. S. Johnson , E. M. Hanks , and J. H. Lowry . 2010. Agent‐based inference for animal movement and selection. Journal of Agricultural, Biological and Environmental Statistics 15:523–538.

[ecy3473-bib-0057] Hooten, M. B. , D. S. Johnson , B. T. McClintock , and J. M. Morales . 2017. Animal movement: statistical models for telemetry data. CRC Press, Boca Raton, Florida, USA.

[ecy3473-bib-0058] Hooten, M. B. , H. R. Scharf , and J. M. Morales . 2019b. Running on empty: recharge dynamics from animal movement data. Ecology Letters 22:377–389. 30548152 10.1111/ele.13198

[ecy3473-bib-0059] Hooten, M. , C. Wikle , and M. Schwob . 2020. Statistical implementations of agent‐based demographic models. International Statistical Review 88:441–461. 32834401 10.1111/insr.12399 PMC7436772

[ecy3473-bib-0060] Horne, J. S. , E. O. Garton , S. M. Krone , and J. S. Lewis . 2007. Analyzing animal movements using Brownian bridges. Ecology 88:2354–2363. 17918412 10.1890/06-0957.1

[ecy3473-bib-0061] Hussey, N. E. , et al. 2015. Aquatic animal telemetry: a panoramic window into the underwater world. Science 348:1255642. 26068859 10.1126/science.1255642

[ecy3473-bib-0062] Johnson, D. H. 1980. The comparison of usage and availability measurements for evaluating resource preference. Ecology 61:65–71.

[ecy3473-bib-0063] Johnson, D. S. , M. B. Hooten , and C. E. Kuhn . 2013. Estimating animal resource selection from telemetry data using point process models. Journal of Animal Ecology 82:1155–1164. 23800202 10.1111/1365-2656.12087

[ecy3473-bib-0064] Johnson, D. S. , J. M. London , M.‐A. Lea , and J. W. Durban . 2008. Continuous‐time correlated random walk model for animal telemetry data. Ecology 89:1208–1215. 18543615 10.1890/07-1032.1

[ecy3473-bib-0065] Jonsen, I. D. , J. M. Flemming , and R. A. Myers . 2005. Robust state–space modeling of animal movement data. Ecology 86:2874–2880.

[ecy3473-bib-0066] Kays, R. , M. C. Crofoot , W. Jetz , and M. Wikelski . 2015. Terrestrial animal tracking as an eye on life and planet. Science 348:aaa2478. 26068858 10.1126/science.aaa2478

[ecy3473-bib-0067] Kidney, D. , B. M. Rawson , D. L. Borchers , B. C. Stevenson , T. A. Marques , and L. Thomas . 2016. An efficient acoustic density estimation method with human detectors applied to gibbons in Cambodia. PLoS One 11:e0155066. 27195799 10.1371/journal.pone.0155066 PMC4873237

[ecy3473-bib-0068] King, R. , et al. 2016. Capture–recapture abundance estimation using a semi‐complete data likelihood approach. Annals of Applied Statistics 10:264–285.

[ecy3473-bib-0069] Kloeden, P. E. , and E. Platen . 1992. Numerical solution of stochastic differential equations. Volume 23. Springer‐Verlag, Berlin, Germany.

[ecy3473-bib-0070] Kranstauber, B. , R. Kays , S. D. LaPoint , M. Wikelski , and K. Safi . 2012. A dynamic Brownian bridge movement model to estimate utilization distributions for heterogeneous animal movement. Journal of Animal Ecology 81:738–746. 22348740 10.1111/j.1365-2656.2012.01955.x

[ecy3473-bib-0071] Lagrange, P. , R. Pradel , M. Bélisle , and O. Gimenez . 2014. Estimating dispersal among numerous sites using capture–recapture data. Ecology 95:2316–2323. 25230481 10.1890/13-1564.1

[ecy3473-bib-0072] Langrock, R. , G. Hopcraft , P. Blackwell , V. Goodall , R. King , M. Niu , T. Patterson , M. Pedersen , A. Skarin , and R. Schick . 2014. Modelling group dynamic animal movement. Methods in Ecology and Evolution 5:190–199.

[ecy3473-bib-0073] Langrock, R. , R. King , J. Matthiopoulos , L. Thomas , D. Fortin , and J. M. Morales . 2012. Flexible and practical modeling of animal telemetry data: hidden Markov models and extensions. Ecology 93:2336–2342. 23236905 10.1890/11-2241.1

[ecy3473-bib-0074] Lebreton, J.‐D. , J. D. Nichols , R. J. Barker , R. Pradel , and J. A. Spendelow . 2009. Modeling individual animal histories with multistate capture–recapture models. Advances in Ecological Research 41:87–173.

[ecy3473-bib-0075] Linden, D. W. , A. P. Sirén , and P. J. Pekins . 2018. Integrating telemetry data into spatial capture–recapture modifies inferences on multi‐scale resource selection. Ecosphere 9:e02203.

[ecy3473-bib-0076] Link, W. A. , J. Yoshizaki , L. L. Bailey , and K. H. Pollock . 2010. Uncovering a latent multinomial: analysis of mark–recapture data with misidentification. Biometrics 66:178–185. 19397581 10.1111/j.1541-0420.2009.01244.x

[ecy3473-bib-0077] Lütkepohl, H. 2013. Introduction to multiple time series analysis. Springer Science & Business Media, Berlin, Germany.

[ecy3473-bib-0078] MacKenzie, D. I. , J. D. Nichols , J. A. Royle , K. H. Pollock , L. Bailey , and J. E. Hines . 2018. Occupancy estimation and modeling: inferring patterns and dynamics of species occurrence. Second edition. Elsevier, San Diego, California, USA.

[ecy3473-bib-0079] Manly, B. , L. McDonald , D. L. Thomas , T. L. McDonald , and W. P. Erickson . 2007. Resource selection by animals: statistical design and analysis for field studies. Springer Science & Business Media, Dordrecht, the Netherlands.

[ecy3473-bib-0080] Maronde, L. , B. T. McClintock , U. Breitenmoser , and F. Zimmermann . 2020. Spatial capture–recapture with multiple noninvasive marks: An application to camera‐trapping data of the European wildcat (*Felis silvestris*) using R package multimark. Ecology and Evolution. 10.1002/ece3.6990 PMC7771165 33391695

[ecy3473-bib-0081] Marques, T. A. , L. Thomas , and J. A. Royle . 2011. A hierarchical model for spatial capture–recapture data: comment. Ecology 92:526–528. 21618931 10.1890/10-1440.1

[ecy3473-bib-0082] Matechou, E. , B. J. Morgan , S. Pledger , J. Collazo , and J. Lyons . 2013. Integrated analysis of capture–recapture–resighting data and counts of unmarked birds at stop‐over sites. Journal of Agricultural, Biological, and Environmental Statistics 18:120–135.

[ecy3473-bib-0083] Matthiopoulos, J. , J. Fieberg , G. Aarts , H. L. Beyer , J. M. Morales , and D. T. Haydon . 2015. Establishing the link between habitat selection and animal population dynamics. Ecological Monographs 85:413–436.

[ecy3473-bib-0084] McCaslin, H. M. , A. B. Feuka , and M. B. Hooten . 2020. Hierarchical computing for hierarchical models in ecology. Methods in Ecology and Evolution. 10.1111/2041-210X.13513

[ecy3473-bib-0085] McClintock, B. T. 2017. Incorporating telemetry error into hidden Markov models of animal movement using multiple imputation. Journal of Agricultural, Biological and Environmental Statistics 22:249–269.

[ecy3473-bib-0086] McClintock, B. T. , P. B. Conn , R. S. Alonso , and K. R. Crooks . 2013. Integrated modeling of bilateral photo‐identification data in mark–recapture analyses. Ecology 94:1464–1471. 23951706 10.1890/12-1613.1

[ecy3473-bib-0087] McClintock, B. T. , D. S. Johnson , M. B. Hooten , J. M. Ver Hoef , and J. M. Morales . 2014. When to be discrete: the importance of time formulation in understanding animal movement. Movement Ecology 2:21. 25709830 10.1186/s40462-014-0021-6 PMC4337762

[ecy3473-bib-0088] McClintock, B. T. , R. King , L. Thomas , J. Matthiopoulos , B. J. McConnell , and J. M. Morales . 2012. A general discrete‐time modeling framework for animal movement using multistate random walks. Ecological Monographs 82:335–349.

[ecy3473-bib-0089] McClintock, B. T. , J. M. London , M. F. Cameron , and P. L. Boveng . 2015. Modelling animal movement using the Argos satellite telemetry location error ellipse. Methods in Ecology and Evolution 6:266–277.

[ecy3473-bib-0090] McClintock, B. T. , and T. Michelot . 2018. momentuHMM: R package for generalized hidden Markov models of animal movement. Methods in Ecology and Evolution 9:1518–1530.

[ecy3473-bib-0091] McLaughlin, P. , and H. Bar . 2020. A spatial capture–recapture model with attractions between individuals. Environmetrics 32:e2653.

[ecy3473-bib-0092] Measey, G. J. , B. C. Stevenson , T. Scott , R. Altwegg , and D. L. Borchers . 2017. Counting chirps: acoustic monitoring of cryptic frogs. Journal of Applied Ecology 54:894–902.

[ecy3473-bib-0093] Mews, S. , R. Langrock , R. King , and N. Quick . 2020. Continuous‐time multi‐state capture‐recapture models. arXiv preprint arXiv:2002.10997.

[ecy3473-bib-0094] Michelot, T. , and P. G. Blackwell . 2019. State‐switching continuous‐time correlated random walks. Methods in Ecology and Evolution 10:637–649.

[ecy3473-bib-0095] Michelot, T. , P. G. Blackwell , and J. Matthiopoulos . 2019a. Linking resource selection and step selection models for habitat preferences in animals. Ecology 100:e02452. 30047993 10.1002/ecy.2452

[ecy3473-bib-0096] Michelot, T. , P. Gloaguen , P. G. Blackwell , and M. P. Étienne . 2019b. The Langevin diffusion as a continuous‐time model of animal movement and habitat selection. Methods in Ecology and Evolution 10:1894–1907.

[ecy3473-bib-0097] Michelot, T. , R. Langrock , and T. A. Patterson . 2016. moveHMM: An R package for the statistical modelling of animal movement data using hidden Markov models. Methods in Ecology and Evolution 7:1308–1315.

[ecy3473-bib-0098] Miller, T. J. , and P. K. Andersen . 2008. A finite‐state continuous‐time approach for inferring regional migration and mortality rates from archival tagging and conventional tag‐recovery experiments. Biometrics 64:1196–1206. 18325073 10.1111/j.1541-0420.2008.00996.x

[ecy3473-bib-0099] Mollet, P. , M. Kéry , B. Gardner , G. Pasinelli , and J. A. Royle . 2015. Estimating population size for capercaillie (*Tetrao urogallus* L.) with spatial capture‐recapture models based on genotypes from one field sample. PLoS ONE 10:e0129020. 26087321 10.1371/journal.pone.0129020 PMC4472805

[ecy3473-bib-0100] Morales, J. M. , D. T. Haydon , J. Frair , K. E. Holsinger , and J. M. Fryxell . 2004. Extracting more out of relocation data: building movement models as mixtures of random walks. Ecology 85:2436–2445.

[ecy3473-bib-0101] Morales, J. M. , P. R. Moorcroft , J. Matthiopoulos , J. L. Frair , J. G. Kie , R. A. Powell , E. H. Merrill , and D. T. Haydon . 2010. Building the bridge between animal movement and population dynamics. Philosophical Transactions of the Royal Society B 365:2289–2301. 10.1098/rstb.2010.0082 PMC2894961 20566505

[ecy3473-bib-0102] Mueller, T. , et al. 2011. How landscape dynamics link individual to population‐level movement patterns: a multispecies comparison of ungulate relocation data. Global Ecology and Biogeography 20:683–694.

[ecy3473-bib-0103] Nathan, R. , W. M. Getz , E. Revilla , M. Holyoak , R. Kadmon , D. Saltz , and P. E. Smouse . 2008. A movement ecology paradigm for unifying organismal movement research. Proceedings of the National Academy of Sciences of the United States of America 105:19052–19059. 19060196 10.1073/pnas.0800375105 PMC2614714

[ecy3473-bib-0104] Nichols, J. D. , and W. L. Kendall . 1995. The use of multi‐state capture–recapture models to address questions in evolutionary ecology. Journal of Applied Statistics 22:835–846.

[ecy3473-bib-0105] Norris, J. R. 1998. Markov chains. Second edition. Cambridge University Press, Cambridge, UK.

[ecy3473-bib-0106] Ovaskainen, O. , H. J. de Knegt , and M. del Mar Delgado . 2016. Quantitative ecology and evolutionary biology: integrating models with data. Oxford University Press, Oxford, UK.

[ecy3473-bib-0107] Ovaskainen, O. 2004. Habitat‐specific movement parameters estimated using mark–recapture data and a diffusion model. Ecology 85:242–257.

[ecy3473-bib-0108] Ovaskainen, O. , and E. E. Crone . 2009. Modeling animal movement with diffusion. Pages 85–106 *in* S. Cantrell , C. Cosner , and S. Ruan , editors. Spatial ecology. Chapman and Hall/CRC, Boca Raton, Florida, USA.

[ecy3473-bib-0109] Ovaskainen, O. , H. Rekola , E. Meyke , and E. Arjas . 2008. Bayesian methods for analyzing movements in heterogeneous landscapes from mark–recapture data. Ecology 89:542–554. 18409443 10.1890/07-0443.1

[ecy3473-bib-0110] Parton, A. , and P. G. Blackwell . 2017. Bayesian inference for multistate ‘step and turn’ animal movement in continuous time. Journal of Agricultural, Biological and Environmental Statistics 22:373–392.

[ecy3473-bib-0111] Patterson, T. A. , A. Parton , R. Langrock , P. G. Blackwell , L. Thomas , and R. King . 2017. Statistical modelling of individual animal movement: an overview of key methods and a discussion of practical challenges. AStA Advances in Statistical Analysis 101:399–438.

[ecy3473-bib-0112] Pedersen, M. W. , T. A. Patterson , U. H. Thygesen , and H. Madsen . 2011. Estimating animal behavior and residency from movement data. Oikos 120:1281–1290.

[ecy3473-bib-0113] Pedersen, M. W. , and K. C. Weng . 2013. Estimating individual animal movement from observation networks. Methods in Ecology and Evolution 4:920–929.

[ecy3473-bib-0114] Pirotta, E. , E. W. J. Edwards , L. New , and P. M. Thompson . 2018. Central place foragers and moving stimuli: A hidden‐state model to discriminate the processes affecting movement. Journal of Animal Ecology 87:1116–1125. 29577275 10.1111/1365-2656.12830

[ecy3473-bib-0115] Potts, J. R. , K. Mokross , and M. A. Lewis . 2014. A unifying framework for quantifying the nature of animal interactions. Journal of the Royal Society Interface 11:20140333. 24829284 10.1098/rsif.2014.0333 PMC4032549

[ecy3473-bib-0116] Potts, J. R. , and U. E. Schlägel . 2020. Parametrising diffusion‐taxis equations from animal movement trajectories using step selection analysis. Methods in Ecology and Evolution 11:1092–1105.

[ecy3473-bib-0117] Preisler, H. K. , A. A. Ager , B. K. Johnson , and J. G. Kie . 2004. Modeling animal movements using stochastic differential equations. Environmetrics 15:643–657.

[ecy3473-bib-0118] Reich, B. J. , and B. Gardner . 2014. A spatial capture–recapture model for territorial species. Environmetrics 25:630–637.

[ecy3473-bib-0119] Roberts, G. O. , and R. L. Tweedie . 1996. Exponential convergence of Langevin diffusions and their discrete approximations. Bernoulli 2:341–363.

[ecy3473-bib-0120] Royle, J. A. , R. B. Chandler , K. D. Gazenski , and T. A. Graves . 2013a. Spatial capture–recapture models for jointly estimating population density and landscape connectivity. Ecology 94:287–294. 23691647 10.1890/12-0413.1

[ecy3473-bib-0121] Royle, J. A. , R. B. Chandler , R. Sollmann , and B. Gardner . 2013b. Spatial capture–recapture. Academic Press, New York, New York, USA.

[ecy3473-bib-0122] Royle, J. A. , R. B. Chandler , C. C. Sun , and A. K. Fuller . 2013c. Integrating resource selection information with spatial capture–recapture. Methods in Ecology and Evolution 4:520–530.

[ecy3473-bib-0123] Royle, J. A. , and R. M. Dorazio . 2012. Parameter‐expanded data augmentation for Bayesian analysis of capture–recapture models. Journal of Ornithology 152:521–537.

[ecy3473-bib-0124] Royle, J. A. , R. M. Dorazio , and W. A. Link . 2007. Analysis of multinomial models with unknown index using data augmentation. Journal of Computational and Graphical Statistics 16:67–85.

[ecy3473-bib-0125] Royle, J. A. , A. K. Fuller , and C. Sutherland . 2016. Spatial capture–recapture models allowing Markovian transience or dispersal. Population Ecology 58:53–62.

[ecy3473-bib-0126] Royle, J. A. , A. K. Fuller , and C. Sutherland . 2018. Unifying population and landscape ecology with spatial capture–recapture. Ecography 41:444–456.

[ecy3473-bib-0127] Royle, J. A. , K. U. Karanth , A. M. Gopalaswamy , and N. S. Kumar . 2009. Bayesian inference in camera trapping studies for a class of spatial capture–recapture models. Ecology 90:3233–3244. 19967878 10.1890/08-1481.1

[ecy3473-bib-0128] Royle, J. A. , M. Kery , and J. Guelat . 2011. Spatial capture–recapture models for search‐encounter data. Methods in Ecology and Evolution 2:602–611.

[ecy3473-bib-0129] Royle, J. A. , and K. V. Young . 2008. A hierarchical model for spatial capture–recapture data. Ecology 89:2281–2289. 18724738 10.1890/07-0601.1

[ecy3473-bib-0130] Scharf, H. , M. B. Hooten , and D. S. Johnson . 2017. Imputation approaches for animal movement modeling. Journal of Agricultural, Biological and Environmental Statistics 22:335–352.

[ecy3473-bib-0131] Schaub, M. , and F. Abadi . 2011. Integrated population models: a novel analysis framework for deeper insights into population dynamics. Journal of Ornithology 152:227–237.

[ecy3473-bib-0132] Schaub, M. , and J. A. Royle . 2014. Estimating true instead of apparent survival using spatial Cormack–Jolly–Seber models. Methods in Ecology and Evolution 5:1316–1326.

[ecy3473-bib-0133] Schwarz, C. J. , J. F. Schweigert , and A. N. Arnason . 1993. Estimating migration rates using tag recovery data. Biometrics 49:177–193.

[ecy3473-bib-0134] Seber, G. A. , and M. R. Schofield . 2019. Capture–recapture: parameter estimation for open animal populations. Springer, Cham, Switzerland.

[ecy3473-bib-0135] Signer, J. , J. Fieberg , and T. Avgar . 2019. Animal movement tools (amt): R package for managing tracking data and conducting habitat selection analyses. Ecology and evolution 9:880–890. 30766677 10.1002/ece3.4823 PMC6362447

[ecy3473-bib-0136] Sollmann, R. , B. Gardner , and J. L. Belant . 2012. How does spatial study design influence density estimates from spatial capture–recapture models? PLoS One 7:e34575. 22539949 10.1371/journal.pone.0034575 PMC3335117

[ecy3473-bib-0137] Sollmann, R. , B. Gardner , A. W. Parsons , J. J. Stocking , B. T. McClintock , T. R. Simons , K. H. Pollock , and A. F. O’Connell . 2013. A spatial mark–resight model augmented with telemetry data. Ecology 94:553–559. 23687880 10.1890/12-1256.1

[ecy3473-bib-0138] Spiegel, O. , S. T. Leu , C. M. Bull , and A. Sih . 2017. What’s your move? Movement as a link between personality and spatial dynamics in animal populations. Ecology Letters 20:3–18. 28000433 10.1111/ele.12708

[ecy3473-bib-0139] Sun, C. C. , A. K. Fuller , and J. A. Royle . 2014. Trap configuration and spacing influences parameter estimates in spatial capture–recapture models. PLoS One 9:e88025. 24505361 10.1371/journal.pone.0088025 PMC3914876

[ecy3473-bib-0140] Tenan, S. , P. Pedrini , N. Bragalanti , C. Groff , and C. Sutherland . 2017. Data integration for inference about spatial processes: A model‐based approach to test and account for data inconsistency. PLoS One 12:e0185588. 28973034 10.1371/journal.pone.0185588 PMC5626469

[ecy3473-bib-0141] Thurfjell, H. , S. Ciuti , and M. S. Boyce . 2014. Applications of step‐selection functions in ecology and conservation. Movement Ecology 2:4. 25520815 10.1186/2051-3933-2-4 PMC4267544

[ecy3473-bib-0142] Thygesen, U. H. , M. W. Pedersen , and H. Madsen . 2009. Geolocating fish using hidden Markov models and data storage tags. Pages 277–293 *in* J. L. Nielsen , H. Arrizabalaga , N. Fragoso , A. Hobday , M. Lutcavage , and J. Sibert , editors. Tagging and tracking of marine animals with electronic devices. Springer, Dordrecht, the Netherlands.

[ecy3473-bib-0143] Tufto, J. , R. Lande , T.‐H. Ringsby , S. Engen , B.‐E. Sæther , T. R. Walla , and P. J. DeVries . 2012. Estimating Brownian motion dispersal rate, longevity and population density from spatially explicit mark–recapture data on tropical butterflies. Journal of Animal Ecology 81:756–769. 22320218 10.1111/j.1365-2656.2012.01963.x

[ecy3473-bib-0144] Wang, Y.‐S. , P. G. Blackwell , J. A. Merkle , and J. R. Potts . 2019. Continuous time resource selection analysis for moving animals. Methods in Ecology and Evolution 10:1664–1678.

[ecy3473-bib-0145] White, G. C. , and K. P. Burnham . 1999. Program MARK: Survival estimation from populations of marked animals. Bird Study 46:S120–S138.

[ecy3473-bib-0146] White, G. C. , and T. M. Shenk . 2001. Population estimation with radio‐marked animals. Pages 329–350 *in* J. Millspaugh , and J. M. Marzluff , editors. Radio tracking and animal populations. Elsevier, San Diego, California, USA.

[ecy3473-bib-0147] Williams, B. K. , J. D. Nichols , and M. J. Conroy . 2002. Analysis and management of animal populations. Academic Press, San Diego, California, USA.

[ecy3473-bib-0148] Williams, P. J. , M. B. Hooten , J. N. Womble , G. G. Esslinger , and M. R. Bower . 2018. Monitoring dynamic spatio‐temporal ecological processes optimally. Ecology 99:524–535. 29369341 10.1002/ecy.2120

[ecy3473-bib-0149] Wilson, K. , E. Hanks , and D. Johnson . 2018. Estimating animal utilization densities using continuous‐time Markov chain models. Methods in Ecology and Evolution 9:1232–1240.

[ecy3473-bib-0150] Winton, M. V. , J. Kneebone , D. R. Zemeckis , and G. Fay . 2018. A spatial point process model to estimate individual centres of activity from passive acoustic telemetry data. Methods in Ecology and Evolution 9:2262–2272.

[ecy3473-bib-0151] Zhao, Q. 2020. On the sampling design of spatially explicit integrated population models. Methods in Ecology and Evolution. 10.1111/2041-210X.13457

