# Supplementary material for: An integrated path for spatial capture–recapture and animal movement modeling
Source: Ecology. 2021 Sep 30;103(10):e3473. doi: 10.1002/ecy.3473 (PMC9786756; doi:10.1002/ecy.3473)
Supplement: Supplementary file 1 — Appendix S1 [file ECY-103-e3473-s001.pdf]

Supporting Information. McClintock, B. T., B. Abrahms, R. B. Chandler, P. B. Conn, S. J. Converse, R. Emmet, B. Gardner, N. J. Hostetter, and D. S. Johnson. 2021. An integrated path for spatial capture-recapture and animal movement modeling. *Ecology*.

Appendix S1: Langevin diffusion RSF with range resident movement behavior

The Langevin diffusion RSF model (Eq. 18 in the main text) could be extended to range resident movement behavior, where each individual has a center of attraction $\mathbf{a}_i$:

$$\mathbf{s}_{it}^* = \begin{cases} \mathbf{s}_{i,t-1} + \frac{\sigma_1^2}{2}\left[\mathbf{D}_0 \nabla c_0\left(\mathbf{s}_{i,t-1}\right) + \sum_{k=1}^K \mathbf{D}_k \nabla c_k\left(\mathbf{s}_{i,t-1}\right)\right] & \text{if } m_i = 1 \text{ (resident)} \\ \mathbf{s}_{i,t-1} + \mathbf{R}\left(\mathbf{s}_{i,t-1} - \mathbf{s}_{i,t-2}\right) & \text{if } m_i = 2 \text{ (transient)} \end{cases}, \tag{S1}$$

where $c_0\left(\mathbf{s}_{i,t-1}\right)$ is the Euclidean distance between $\mathbf{a}_i$ and $\mathbf{s}_{i,t-1}$. The individual-level utilization distribution for residents (arising from "third-order" resource selection within a home range; e.g., Johnson, 1980; Royle et al., 2018) is then

$$\pi\left(\mathbf{s} \mid \mathbf{a}_i\right) = \frac{\exp\left(\delta_0 \|\mathbf{a}_i - \mathbf{s}\| + \sum_{k=1}^K \delta_k c_k\left(\mathbf{s}\right)\right)}{\int_{\mathcal{M}} \exp\left(\delta_0 \|\mathbf{a}_i - \mathbf{z}\| + \sum_{k=1}^K \delta_k c_k\left(\mathbf{z}\right)\right) d\mathbf{z}}, \tag{S2}$$

which could be used for the point process model of the initial locations:

$$\left[\mathbf{s}_{i1} \mid m_i, \mathbf{a}_i, \boldsymbol{\theta}\right] = \begin{cases} \pi\left(\mathbf{s}_{i1} \mid \mathbf{a}_i\right) & \text{if } m_i = 1 \text{ (resident)} \\ \text{Uniform}\left(\mathcal{M}\right) & \text{if } m_i = 2 \text{ (transient)} \end{cases}.$$

Assuming the centers of attraction are uniformly distributed, Eq. 19 would be the simplest approach to deriving a population-level utilization distribution, in which case the population-level utilization distribution would be

$$\pi(\mathbf{s}) = \int_{\mathcal{M}} \pi(\mathbf{s} \mid \mathbf{a}) d\mathbf{a}.$$

For inhomogeneously-distributed centers of attraction, "second-order" resource selection (i.e., individual home range selection within $\mathcal{M}$) could be investigated by including a point process model describing spatial variation in the density of centers of attraction (as in Eq. 14), and the population utilization distribution would be

$$\pi(\mathbf{s}) = \int_{\mathcal{M}} \pi(\mathbf{s} \mid \mathbf{a}) \left[\mathbf{a} \mid \boldsymbol{\theta}\right] d\mathbf{a}.$$

For example, under an inhomogeneous Poisson point process model with center of attraction density surface $[\mathbf{a}_i \mid m_i = 1, \boldsymbol{\theta}] = \frac{\lambda(\mathbf{a}_i)}{\int_{\mathcal{M}} \lambda(\mathbf{z}) d\mathbf{z}}$, we could include the same covariates as for third-order selection, i.e., $\lambda(\mathbf{a}_i) = \exp\left(\sum_{k=1}^{K} \beta_k c_k(\mathbf{a}_i)\right)$, or include alternative covariates describing second-order selection.

Literature Cited

Johnson, D. H. 1980. The comparison of usage and availability measurements for evaluating resource preference. Ecology, **61**:65–71.

Royle, J. A., A. K. Fuller, and C. Sutherland. 2018. Unifying population and landscape ecology with spatial capture–recapture. Ecography, **41**:444–456.
